# Condition-Specific Growth Charts for Children With Alagille Syndrome

**DOI:** 10.1001/jamanetworkopen.2025.45294

**Published:** 2025-11-24

**Authors:** Koen Huysentruyt, Shannon M. Vandriel, Mathieu Roelants, David A. Piccoli, Kathleen M. Loomes, Elizabeth B. Rand, Noelle H. Ebel, Jeffrey A. Feinstein, Irena Jankowska, Piotr Czubkowski, Dorota Gliwicz-Miedzińska, Emmanuel M. Gonzales, Emmanuel Jacquemin, Jérôme Bouligand, Saul J. Karpen, Rene Romero, Henry C. Lin, Björn Fischler, Henrik Arnell, Li-Ting Li, Jian-She Wang, Rima Fawaz, Silvia Nastasio, Kyung Mo Kim, Seak Hee Oh, Lorenzo D’Antiga, Emanuele Nicastro, Ryan T. Fischer, Susan M. Siew, Michael Stormon, Chatmanee Lertudomphonwanit, Winita Hardikar, Sahana Shankar, James E. Squires, Shikha S. Sundaram, Catherine Larson-Nath, Deirdre A. Kelly, Jane Hartley, Pinar Bulut, M. Kyle Jensen, Catalina Jaramillo, Amin J. Roberts, Helen M. Evans, Étienne M. Sokal, Tanguy Demaret, Henkjan J. Verkade, Richard J. Thompson, Bettina E. Hansen, Tim J. Cole, Binita M. Kamath, and the Global Alagille Alliance (GALA) Study Group, Dominique Debray, Florence Lacaille, Jernej Brecelj, Nehal M. El-Koofy, Mohamed A. Elmonem, Way Seah Lee, Maria Camila Sanchez, Maria Lorena Cavalieri, Christina Hajinicolaou, Kathleen B. Schwarz, Elisa Carvalho, Nathalie Rock, Wikrom Karnsakul, Ruben E. Quiros-Tejeira, Seema Alam, Gabriella Nebbia, Yael Mozer-Glassberg, Pamela L. Valentino, Ermelinda Santos-Silva, Zerrin Önal, Antal Dezsőfi-Gottl, Melina Melere, María Legarda Tamara, John Eshun, Aglaia Zellos, Giuseppe Indolfi, Maria Rogalidou, Niviann Blondet, Pier Luigi Calvo, Marisa Beretta, Andréanne N. Zizzo, Cigdem Arikan, Mureo Kasahara, Nanda Kerkar, Amal A. Aqul, Victorien M. Wolters, Raquel Borges Pinto, Jesus Quintero Bernabeu, Jennifer Garcia, Sabina Wiecek, Christos Tzivinikos, Quais Mujawar, Carolina Jimenez-Rivera, Cristina Molera Busoms, Cristina Gonçalves, Luis Bujanda

**Affiliations:** 1 Kindergastro-enterologie, hepatologie en nutritie, UZ Brussel, Vrije Universiteit Brussel, Brussels, Belgium; 2 Division of Gastroenterology, Hepatology and Nutrition, The Hospital for Sick Children and the University of Toronto, Toronto, Ontario, Canada; 3 Environment and Health, Department of Public Health and Primary Care, KU Leuven, Leuven, Belgium; 4 Division of Gastroenterology, Hepatology and Nutrition, The Children’s Hospital of Philadelphia and the University of Pennsylvania Perelman School of Medicine, Philadelphia; 5 Division of Gastroenterology, Department of Pediatrics, Stanford University School of Medicine, Palo Alto, California; 6 Department of Pediatrics (Cardiology), Stanford University School of Medicine, Lucile Packard Children’s Hospital, Palo Alto, California; 7 Department of Gastroenterology, Hepatology, Nutrition Disturbances and Pediatrics, The Children’s Memorial Health Institute, Warsaw, Poland; 8 Pediatric Hepatology and Liver Transplantation Unit, National Reference Centre for Rare Pediatric Liver Diseases, French Network for Rare Liver Disease, European Reference Network on Hepatological Diseases, Bicêtre Hospital, Assistance Publique–Hȏpitaux de Paris, Université Paris-Saclay, Le Kremlin-Bicêtre, France; 9 Inserm U1193, Hepatinov, University of Paris-Saclay, Orsay, France; 10 Service de Génétique Moléculaire, Pharmacogénétique et Hormonologie, Hôpitaux Universitaires Paris-Saclay, Assistance Publique-Hôpitaux de Paris, Centre Hospitalier Universitaire de Bicêtre, Le Kremlin-Bicêtre, France; 11 Division of Pediatric Gastroenterology, Hepatology and Nutrition, Children’s Healthcare of Atlanta, Emory University School of Medicine, Atlanta, Georgia; 12 Stravitz-Sanyal Institute for Liver Disease and Metabolic Health at Virginia Commonwealth University, Richmond; 13 Division of Pediatric Gastroenterology, Department of Pediatrics, Oregon Health and Science University, Portland; 14 Department of Paediatric Gastroenterology, Hepatology and Nutrition, Astrid Lindgren Children’s Hospital, Karolinska University Hospital, CLINTEC, Karolinska Institutet, European Reference Network on Hepatological Diseases, Stockholm, Sweden; 15 Department of Paediatric Gastroenterology, Hepatology and Nutrition, Astrid Lindgren Children’s Hospital, Karolinska University Hospital, Department of Women’s and Children’s Health, Karolinska Institutet, European Reference Network on Hepatological Diseases, Stockholm, Sweden; 16 The Center for Pediatric Liver Diseases, Children’s Hospital of Fudan University, Shanghai, China; 17 Department of Pediatrics, Yale University School of Medicine, New Haven, Connecticut; 18 Division of Gastroenterology, Hepatology, and Nutrition, Boston Children’s Hospital and Harvard Medical School, Boston, Massachusetts; 19 Department of Pediatrics, University of Ulsan College of Medicine, Asan Medical Center Children’s Hospital, Seoul, Republic of Korea; 20 Pediatric Hepatology, Gastroenterology and Transplantation, Ospedale Papa Giovanni XXIII, Bergamo, Italy; 21 Department of Medicine and Surgery, University of Milano–Bicocca, Milan, Italy; 22 Section of Hepatology, Department of Gastroenterology, Children’s Mercy Kansas City, Kansas City, Missouri; 23 Department of Gastroenterology, The Children’s Hospital at Westmead, Sydney, New South Wales, Australia; 24 Division of Gastroenterology, Department of Pediatrics, Ramathibodi Hospital Mahidol University, Bangkok, Thailand; 25 Department of Gastroenterology and Clinical Nutrition, Royal Children’s Hospital, Melbourne, Victoria, Australia; 26 Mazumdar Shaw Medical Center, Narayana Health, Bangalore, India; 27 Division of Pediatric Gastroenterology and Hepatology, Department of Pediatrics, University of Pittsburgh School of Medicine, Pittsburgh, Pennsylvania; 28 Section of Gastroenterology, Hepatology and Nutrition, Department of Pediatrics and the Digestive Health Institute, Children’s Hospital of Colorado and University of Colorado School of Medicine, Aurora; 29 Division of Pediatric Gastroenterology, Hepatology, and Nutrition, University of Minnesota, Minneapolis; 30 Liver Unit, Birmingham Women’s and Children’s Hospital, NHS Trust, and University of Birmingham, Birmingham, United Kingdom; 31 Division of Pediatric Gastroenterology and Hepatology, Phoenix Children’s Hospital, Phoenix, Arizona; 32 Division of Pediatric Gastroenterology, Hepatology and Nutrition, Primary Children’s Hospital, University of Utah, Salt Lake City; 33 Department of Paediatric Gastroenterology, Starship Child Health, Auckland, New Zealand; 34 Cliniques Universitaires Saint-Luc, Service De Gastroentérologie et Hépatologie Pédiatrique, Brussels, Belgium; 35 Department of Pediatrics, University Medical Center Groningen, University of Groningen, Groningen, the Netherlands; 36 Institute of Liver Studies, King’s College London, London, United Kingdom; 37 Toronto General Hospital University Health Network, Toronto, Ontario, Canada; 38 Institute of Health Policy, Management and Evaluation, Toronto, Ontario, Canada; 39 UCL Great Ormond Street Institute of Child Health, London, United Kingdom; 40 Pediatric Liver Unit, National Reference Centre for Rare Pediatric Liver Diseases (Biliary Atresia and Genetic Cholestasis), French Network for Rare Liver Disease, European Reference Network on Hepatological Diseases, Necker-Enfants Malades Hospital, University of Paris, Paris, France; 41 Pediatric Gastroenterology-Nutrition and Hepatology Units, Necker-Enfants Malades Hospital, University of Paris, Paris, France; 42 Pediatric Gastroenterology, Hepatology and Nutrition and Department of Pediatrics, Faculty of Medicine, University Medical Center Ljubljana, Ljubljana, Slovenia; 43 Department of Pediatrics, Faculty of Medicine, Cairo University, Cairo, Egypt; 44 Department of Clinical and Chemical Pathology, Faculty of Medicine, Cairo University, Cairo, Egypt; 45 Faculty of Medicine, Department of Paediatrics, University of Malaya, Kuala Lumpur, Malaysia; 46 Pediatric Gastroenterology and Hepatology Division, Hospital Italiano Buenos Aires, Buenos Aires, Argentina; 47 Division of Paediatric Gastroenterology, Chris Hani Baragwanath Academic Hospital, Department of Paediatrics and Child Health, University of the Witwatersrand, Johannesburg, South Africa; 48 Rady Children’s Hospital San Diego, Division of Pediatric Gastroenterology, University of California, San Diego; 49 Pediatric Gastroenterology Department, Hospital da Crianca de Brasilia, Centro Universitario de Brasilia, Brasilia, Federal District, Brazil; 50 Swiss Pediatric Liver Center, Division of Pediatric Specialties, Department of Pediatrics, Gynecology, and Obstetrics, University Hospitals Geneva and University of Geneva, Geneva, Switzerland; 51 Department of Pediatrics, Johns Hopkins University School of Medicine, Baltimore, Maryland; 52 Department of Pediatrics, Children’s Nebraska and University of Nebraska Medical Center, Omaha; 53 Department of Pediatric Hepatology, Institute of Liver and Biliary Sciences, New Delhi, India; 54 Fondazione IRCCS Ca’ Granda Ospedale Maggiore Policlinico, Servizio di Epatologia Pediatrica, Milan, Italy; 55 Institute of Gastroenterology, Nutrition and Liver Diseases, Schneider Children’s Medical Center of Israel, Petah Tikva, Israel; 56 Gastroenterology and Hepatology Division, Department of Pediatrics, University of Washington, Seattle Children’s Hospital, Seattle; 57 Centro Hospitalar Universitario de Santo Antonio, Centro Materno-Infantil do Norte Albino Aroso, Porto, Portugal; 58 UCIBIO–Applied Molecular Biosciences Unit, Biochemistry Laboratory, Department of Biological Sciences, Faculdade de Farmacia, Universidade do Porto, Porto, Portugal; 59 Pediatric Gastroenterology, Hepatology and Nutrition Department, Istanbul University Faculty of Medicine, Istanbul, Turkey; 60 First Department of Paediatrics, Semmelweis University, Budapest, Hungary; 61 Pediatric Gastroenterology Service, Hospital da Crianca Santo Antonio, Universidade Federal de Ciencias da Saude de Porto Alegre, Complexo Hospitalar Santa Casa, Porto Alegre, Rio Grande do Sul, Brazil; 62 Paediatric Gastroenterology Unit, Cruces University Hospital, Bilbao, Spain; 63 Department of Pediatric Gastroenterology, Le Bonheur Children’s Hospital, The University of Tennessee Health Science Center, Memphis; 64 Mitera Children’s Hospital, Athens, Greece; 65 Meyer Children’s University Hospital IRCCS, Florence, Italy; 66 Division of Gastroenterology and Hepatology, “Aghia Sofia” Children’s Hospital, First Department of Pediatrics, University of Athens, Athens, Greece; 67 Pediatric Gastroenterology Unit, Regina Margherita Children’s Hospital, Azienda Ospedaliera-Universitaria Citta’ della Salute e della Scienza, Turin, Italy; 68 Faculty of Health Sciences, Wits Donald Gordon Medical Centre, University of the Witwatersand, Johannesburg, South Africa; 69 Children’s Hospital, London Health Sciences Centre, Division of Paediatric Gastroentereology and Hepatology, Western University, London, Ontario, Canada; 70 Department of Pediatric Gastroenterology and Organ Transplant, Koc University School of Medicine, Istanbul, Turkey; 71 Organ Transplantation Center, National Center for Child Health and Development, Tokyo, Japan; 72 Massachusetts General Hospital for Children, Boston; 73 University of Rochester Medical Center, Rochester, New York; 74 University of Texas Southwestern Medical Center and Children’s Health, Dallas; 75 Department of Pediatric Gastroenterology, University Medical Center Utrecht, Utrecht, the Netherlands; 76 Division of Pediatric Gastroenterology, Hospital Crianca Conceicao, Grupo Hospitalar Conceicao, Porto Alegre, Rio Grande do Sul, Brazil; 77 Pediatric Hepatology and Liver Transplant Department, Hospital Universiatri Vall d’Hebron, Barcelona, Spain; 78 European Reference Network on Hepatological Diseases, Fondazione IRCCS San Gerardo dei Tintori Hospital, Monza, Italy; 79 European Reference Network on Transplantation in Children, La Paz Institute of Biomedical Research, Hospital Universitario La Paz, Madrid, Spain; 80 Division of Pediatric Gastroenterology, Hepatology and Nutrition, Miami Transplant Institute, University of Miami, Miami, Florida; 81 Department of Pediatrics, Medical University of Silesia in Katowice, Katowice, Poland; 82 Department of Paediatric Gastroenterology, Al Jalila Children’s Specialty Hospital, Mohammed Bin Rashid University of Medicine and Health Sciences, Dubai, United Arab Emirates; 83 Section of Pediatric Gastroenterology, Department of Pediatrics, University of Manitoba, Winnipeg, Canada; 84 Division of Gastroenterology, Hepatology and Nutrition, Children’s Hospital of Eastern Ontario, Ottawa, Canada; 85 Pediatric Gastroenterology Hepatology and Nutrition Unit, Hospital Sant Joan de Deu, Esplugues de Llobregat, Spain; 86 Pediatric Gastroenterology/Hepatology Center, European Reference Network on Hepatological Diseases, Lisbon, Portugal; 87 Department of Hepatology and Gastroenterology, Biodonostia Health Research Institute–Donostia University Hospital, Universidad del Pais Vasco, Centro de Investigacion Biomedica en Red de Enfermedades Hepaticas y Digestivas, San Sebastian, Spain

## Abstract

**Importance:**

Different degrees of growth delay have been reported in children with Alagille syndrome (ALGS), yet these patients are routinely evaluated using standard growth charts.

**Objective:**

To develop condition-specific growth charts for ALGS using modern statistical approaches.

**Design, Setting, and Participants:**

This case series used data from the international, multicenter Global Alagille Alliance (GALA) study accrued between May 14, 2018, and March 20, 2023. Children born at full term between January 1, 1997, and August 31, 2019, with a clinically and/or genetically confirmed ALGS diagnosis and their native liver were included. Data from children with a known history of prematurity were excluded for the development of the growth charts. Data were analyzed from March 25, 2023, to December 30, 2024.

**Exposure:**

Growth of children with Alagille syndrome.

**Main Outcomes and Measures:**

Generalized additive models for location scale and shape were fitted to generate percentile plots for weight and height relative to age and superimposed on US Centers for Disease Control and Prevention (CDC) growth charts to illustrate differences in growth patterns compared with children with typical development.

**Results:**

Data from 1204 children with ALGS in overlapping cohorts (median [IQR] gestational age, 38 [37-39] weeks) were analyzed (1204 in the weight cohort; 695 boys [57.7%]; 9855 weight observations; 995 with neonatal cholestasis [82.6%]; 306 receiving a liver transplant [25.4%]; 98 deaths [8.1%] and 1106 in the height cohort, 635 boys [57.4%]; 8464 height observations; 906 with neonatal cholestasis [81.9%]; 287 receiving a liver transplant [25.9%]; 86 deaths [7.8%]) were included for the modeling of the weight-for-age and height-for-age charts, respectively. The median birth weight was 2.8 kg (IQR, 2.5-3.0 kg) for boys and 2.6 kg (IQR, 2.4-2.9 kg) for girls. The median birth length was 48.0 cm (IQR, 46.0-50.0 cm) for boys and 47.0 cm (IQR, 45.0-49.0 cm) for girls. The weight-for-age and height-for-age growth charts for boys and girls with AGLS differed significantly from CDC growth charts. The estimated height at age 18 years corresponded to the 50th percentile was 171.5 cm for boys and 156.5 cm for girls on the condition-specific charts vs 176 cm and 163 cm, respectively, on the CDC growth charts.

**Conclusions and Relevance:**

These findings suggest that condition-specific growth charts for ALGS may provide a crucial tool for clinicians to evaluate growth and aid in decision-making around listing children for liver transplant.

## Key Points

**Question:**

What do condition-specific growth charts look like for children with Alagille syndrome (ALGS)?

**Findings:**

In this case series of 1204 children with ALGS, growth charts developed for boys and girls using modern statistical techniques based on 9855 weight and 8463 height observations showed markedly different growth patterns when overlaid with US growth charts for children with typical development.

**Meaning:**

These findings suggest that condition-specific growth charts may provide references for the growth of children with ALGS and how the condition responds to new therapies.

## Teaser

This case series examines weight and height growth patterns in children with Alagille syndrome and uses modern statistical approaches to develop condition-specific growth charts for these children.

## Introduction

Alagille syndrome (ALGS) is a rare, multiorgan disease caused by a mutation in the *JAG1* or *NOTCH2* genes.^1^ Variable degrees of growth impairment have been reported in children with ALGS.^2,3,4^ The etiology for this growth delay is multifactorial and includes cholestatic liver disease, cardiac involvement, and intrinsic genetic effects.^5^ Severity of cholestasis (as determined by total bilirubin levels) was recently reported to be negatively associated with weight and height in a prospective cohort of children with ALGS.^3^ Cholestasis is associated with fat malabsorption and increased energy and macronutrient needs,^6,7^ despite that the resting energy metabolism of children with ALGS is no different than that of children with typical development.^2,8^ An increase in height *z* scores was noted in children with ALGS and intractable pruritus treated with maralixibat, an apical, sodium-dependent, bile acid transport inhibitor that ameliorates cholestasis by lowering serum bile acids.^9^ The study lacked a control group, however, making it difficult to distinguish between a true effect on growth and the natural height evolution in children with ALGS.

An analysis of growth parameters of 91 children with ALGS found more pronounced catch-up growth, especially in the first 2 years after liver transplant, than in children who were transplanted for biliary atresia. Despite this more pronounce catch-up growth in children with ALGS vs bilateral atresia, final height in the ALGS group remained substantially lower than in the biliary atresia group,^10^ suggesting that the growth deficit might be, at least partially, inherent to the genetic underpinnings of ALGS. A more pronounced deficit in height, as well as in mid–upper-arm circumference, in school-aged children with ALGS compared with age- and sex-matched children with biliary atresia has also been previously described.^4^

The role of cardiac defects in growth impairment in ALGS has been recognized, which may also be associated with decreased energy and macronutrient intake in this subpopulation.^7^ Furthermore, higher levels of circulating growth hormone (GH) were found in a small study of children with cholestasis, suggesting GH resistance in these children.^11^ This hypothesis seems plausible as a follow-up study found no hormonal response to GH administration in 4 children with ALGS and short stature.^12^

Linear growth is a commonly accepted indicator of health during childhood. Since growth in children with ALGS differs from children with typical development, condition-specific growth charts are necessary to help clinicians understand the growth patterns of patients with ALGS and better interpret the impact on growth of liver transplant or new therapies. Furthermore, to ensure a general use of the condition-specific growth charts, children with ALGS from different parts of the world should be included when constructing them to overcome the issue of variations in growth trajectories in different countries.

Therefore, the primary aim of this study was to develop condition-specific growth charts for children with ALGS and their native liver. As secondary aims, the study investigated the performance of the condition-specific growth charts in a subgroup of children with at least 10 years of follow-up to compare the condition-specific growth charts with those of children with typical development and to describe the anthropometric characteristics at birth of children with ALGS.

## Methods

This case series used data from the Global Alagille Alliance (GALA) study, an international, multicenter retrospective study of children with a clinically and/or genetically confirmed ALGS diagnosis born between January 1, 1997, and August 31, 2019. A total of 69 centers participated, including 3 in Africa, 6 in Asia, 25 in Europe, 4 in the Middle East, 24 in North America, 3 in Oceania, and 4 in South America. The ethics committee at each participating center approved the study, or an exemption from ethics approval was granted in accordance with institutional regulations. The ethics committees waived informed consent because the data were retrospective. This report follows reporting guidelines for case series studies.

More details about the study design and data collection have previously been described.^13^ In brief, data were extracted from clinic visits or inpatient hospitalizations until the patient underwent a liver transplant, was transferred to another center, was enrolled in a clinical drug trial for cholestasis-induced pruritus, or died. Data were accrued between May 14, 2018, and March 20, 2023, and analyzed from March 25, 2023, to December 30, 2024.

For the baseline description of the cohort, *z* scores for birth weight and length were calculated and corrected for gestational age using the revised Fenton growth charts.^14^ Data points prior to liver transplant only were included. Gestational age was expressed as the number of completed weeks of gestation. Children with birth weight below the 10th percentile were considered small for gestational age (SGA). The association of birth parameters (prematurity, birth length, and birth weight) with the following clinical phenotype parameters was investigated: year of birth, genetic variant, presence of neonatal cholestasis, presence of characteristic facies, the presence of a cardiac anomaly requiring intervention, the presence of butterfly vertebrae, the presence of dysplastic kidneys, and liver transplant listing later in life. When multiple comparisons were performed, a Holm correction was used to calculate the *P* values.^15^

Data from children with a known history of prematurity (gestational age <37 weeks) were excluded for the development of the growth charts (eFigure 1 in Supplement 1). For weight and height measurements obtained from short hospitalizations, only the measurements closest to admission and discharge were retained; for admissions lasting longer than 1 month, weekly or monthly measurements were retained, depending on the age of the child. Individual curves of weight for age (WFA) and height for age (HFA) were plotted for each patient to assess plausibility of the data. Given the design of the GALA study, in which anthropometric data from every health care contact were collected, hospitalized children were expected and observed to be heavily overrepresented. Time intervals were created to obtain a more balanced dataset (intervals of 1 month for the first 2 years of life, every 3 months for 2-5 years, and every 6 months until adulthood). The first available weight and height measurement from each time interval was used to create the balanced dataset used for the growth chart development. The difference in follow-up time and number of data points between the original (unbalanced) and balanced dataset is presented in eTable 1 in Supplement 1. Different datasets were created for sensitivity analyses: (1) a dataset in which the last measurement from each time interval was used and (2) the original dataset in which each observation was given a weight, calculated as 1/n, with n being the number of available weight or height measurements per predefined time interval.

### Statistical Analysis

The percentile charts were created using generalized additive models for location scale and shape via the gamlss package in R, version 4.0.3 (R Foundation for Statistical Computing).^16^ The best-performing distributions in this work were the Box-Cox Cole and Green (BCCG) and the Box-Cox power exponential (BCPE) distribution. The BCCG is summarized by its 3 parameters median, coefficient of variation, and skewness, while a fourth (kurtosis) is specified in the BCPE distribution. The median, coefficient of variation, skewness, and kurtosis parameters were modeled consecutively, as these processes can be done relatively independently from one another, while visually confirming that no undesired behavior of the percentile lines was observed.^17^ Modeling was done using observations until age 20 years, but the charts were truncated at age 18 years. Model selection was based on the generalized Akaike information criterion with a penalty term of 3. The best-performing models were further evaluated using worm plots.^18^ Height models fitted best with BCCG and weight models with BCPE. Models were summarized in LMS tables, where L (λ) represents the skewness, M the median, and S the coefficient of variation of the data. Since adjusting for kurtosis affects only the most extreme percentiles,^19^ preference was given to BCCG models for the sake of simplicity in calculations of *z* scores. Model validation was done by an overlay plot of the percentiles created by the final model on the 3 different aforementioned datasets. Finally, a subgroup of children with at least 10 years of follow-up was selected to investigate their longitudinal growth on the condition-specific weight and height charts. We have added to the growth charts a macro programmed using Excel, version 2507 (Microsoft Corporation) that easily allows calculations of *z* scores for skinfolds, mid–upper-arm circumference, and our condition-specific height and weight growth charts to promote a more complete nutritional assessment and avoid unnecessary nutritional interventions (Supplement 2). Linear mixed modeling was used to explore the evolution of weight and height *z* scores over time. Variables involved in interaction terms were centered. *P* < .05 was considered statistically significant.

## Results

### Baseline Description Population for Growth Chart Development

Data from 1204 children with ALGS in overlapping cohorts (median [IQR] gestational age, 38 [37-39] weeks) were analyzed, including 1204 for the weight charts (695 boys [57.7%] and 509 girls [42.3%]) and 1106 for the height charts (635 boys [57.4%] and 471 girls [42.6%]). This analysis included children from 71 centers in 30 countries. More details about the follow-up time per patient and number of measurements per age interval are provided in eTables 1 and 2 in Supplement 1. The number of observations per age interval decreased from birth to adulthood. An overview of the baseline characteristics of patients is presented in the Table. The majority had a history of neonatal cholestasis (weight cohort, 995 [82.6%]; height cohort, 906 [81.9%]), and 306 children [25.4%] in the weight cohort and 287 [25.9%] in the height cohort required a liver transplant. Causes of death for 98 children were from liver disease (17 [1.4%]), cardiac disease (16 [1.3%]), multiorgan failure (15 [1.2%]), noncardiac vascular complications (15 [1.2%]), sepsis (13 [1.1%]), liver transplant complication (8 [0.6%]), bleeding (6 [0.5%]) and other or unknown cause (8 [0.6%]). A total of 349 children in the weight cohort (29.0%) and 328 in the height cohort (29.7%) received parenteral and/or enteral nutrition at home at some point.

**Table.** Baseline Characteristics

Characteristic	Children, No. (%)	
	Weight cohort (n = 1204)	Height cohort (n = 1106)
Genotype		
* JAG1*	836 (69.4)	778 (70.3)
* NOTCH2*	31 (2.6)	28 (2.5)
Unknown or negative	337 (28.0)	300 (27.1)
Neonatal cholestasis^a^	995 (82.6)	906 (81.9)
Characteristic facies^b^	1028 (85.4)	941 (85.1)
Posterior embryotoxon^c^	524 (43.5)	485 (43.9)
Butterfly vertebrae^d^	462 (38.4)	414 (37.4)
Cardiac anomaly needing intervention	294 (24.4)	279 (25.2)
Kidney dysplasia^e^	66 (5.5)	62 (5.6)
Total parenteral and/or enteral feeding	349 (29.0)	328 (29.7)
Death	98 (8.1)	86 (7.8)
Age at death, median (IQR), y	2.7 (1.2-4.8)	3.0 (1.5-5.7)
Liver transplant	306 (25.4)	287 (25.9)
Age at liver transplant, median (IQR), y	2.9 (1.6-5.9)	4.5 (1.6-5.9)

^a^ Not known or registered in 34 patients from the weight cohort and 33 from the height cohort.

^b^ Not known or registered in 65 patients from the weight cohort and 61 from the height cohort.

^c^ Not known or registered in 195 patients from the weight cohort and 173 from the height cohort.

^d^ Not known or registered in 132 patients from the weight cohort and 126 from the height cohort.

^e^ Not known or registered in 116 patients from the weight cohort and 98 from the height cohort.

### Birth History

Gestational age at birth was reported for 911 children with ALGS (519 boys [57.0%] and 392 girls [43.0%] girls) from Africa (48 [5.6%]), Asia (93 [10.2%]), Europe (311 [34.1%]), the Middle East (40 [4.4%]), North America (352 [38.6%]), Oceania (31 [3.4%]), and South America (36 [4.0%]). Overall, 179 children (19.6%) were born premature (no significant sex difference). The proportion of premature births was significantly different across regions, being most frequent in Oceania (410 [45.0%]) and least frequent in Africa (91 [10.0%]) (*P* = .002). The children born prematurely were not included in the growth chart development.

Median gestational age–corrected birth weight (701 children [58.2%]) and birth length (357 children [29.7%]) *z* scores were −1.32 (IQR, −1.95 to −0.73) and −0.87 (IQR −1.85 to −0.22) (*P* < .001). Birth weight was classified as SGA for 362 children (51.6%). There was no statistically significant difference between sexes in birth gestational age–corrected *z* scores or SGA occurrence. The median birth weight was 2.8 kg (IQR, 2.5-3.0 kg) for boys and 2.6 kg (IQR, 2.4-2.9 kg) for girls. The median birth length was 48.0 cm (IQR, 46.0-50.0 cm) for boys and 47.0 cm (IQR, 45.0-49.0 cm) for girls. The distributions of birth weights (361 boys [56.3%] and 280 girls [43.7%]) and lengths (172 boys [55.0%] and 141 girls [45.0%]) of the children born at full term are presented in eFigure 2 in Supplement 1. Median birth weights were not significantly different according to any of the clinical phenotypes. Median birth length was significantly lower in children with a dysplastic kidney (45.0 [IQR, 43.5-48.1] vs 48.0 [IQR, 46.0-49.5] cm; *P* = .007); no association was found with any of the other clinical phenotypes.

### Condition-Specific Growth Charts for ALGS

**Figure 1.**
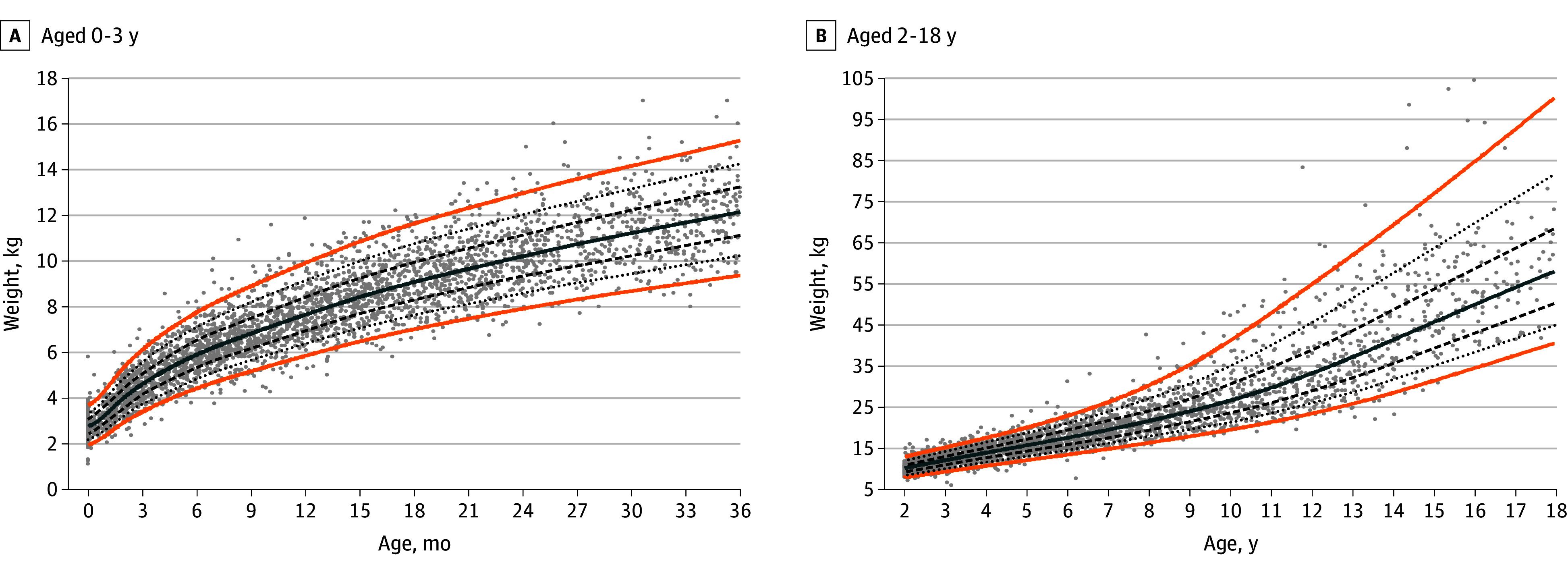
Condition-Specific Weight-for-Age Charts for Boys With Alagille Syndrome Orange lines indicate the third and 97th percentiles; solid blue line, the 50th percentile; dashed lines, the 25th and 75th percentiles; dotted lines, the 10th and 90th percentiles; and dots, observations.

**Figure 2.**
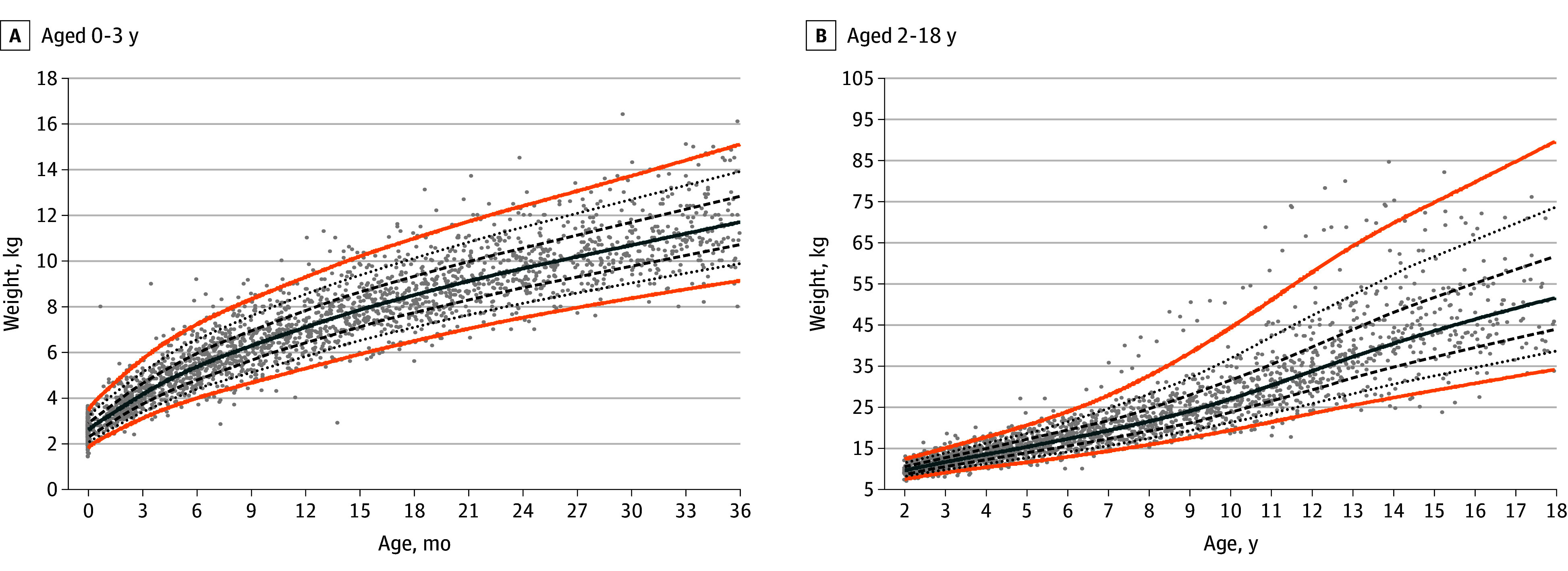
Condition-Specific Weight-for-Age Charts for Girls With Alagille Syndrome Orange lines indicate the third and 97th percentiles; solid blue line, the 50th percentile; dashed lines, the 25th and 75th percentiles; dotted lines, the 10th and 90th percentiles; and dots, observations.

**Figure 3.**
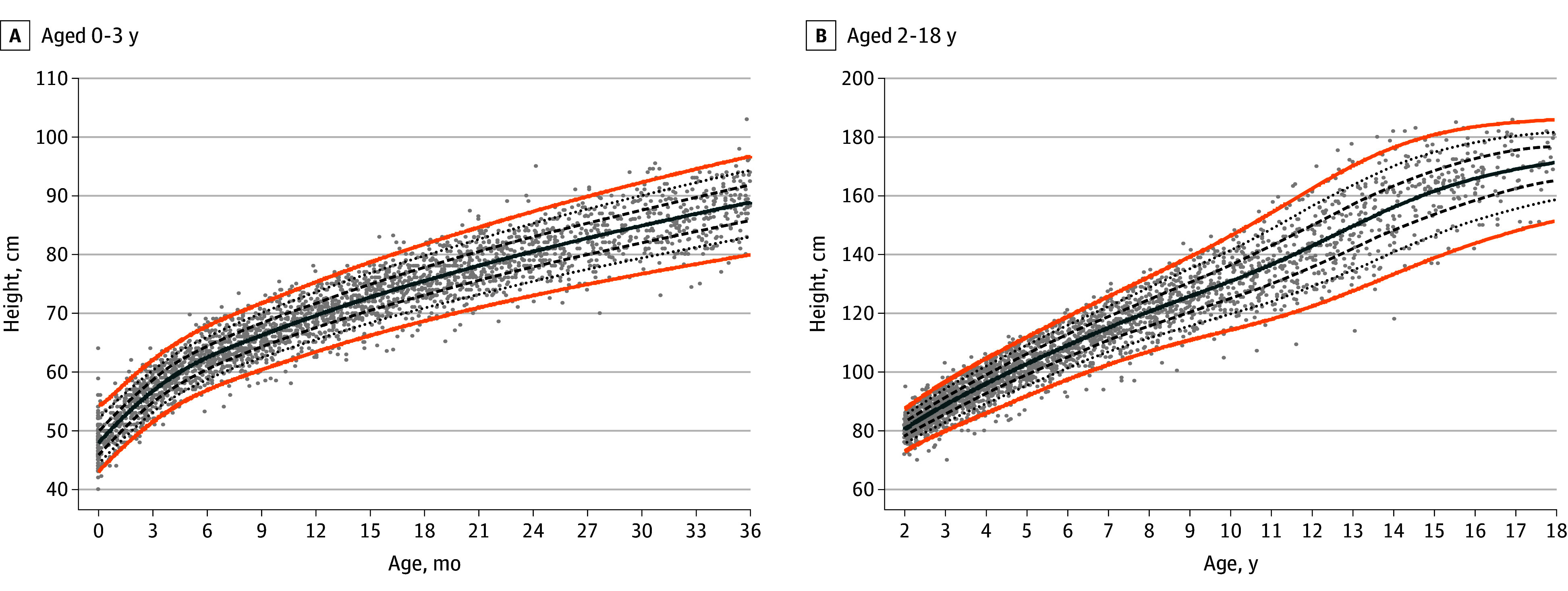
Condition-Specific Height-for-Age Charts for Boys With Alagille Syndrome Orange lines indicate the third and 97th percentiles; solid blue line, the 50th percentile; dashed lines, the 25th and 75th percentiles; dotted lines, the 10th and 90th percentiles; and dots, observations.

**Figure 4.**
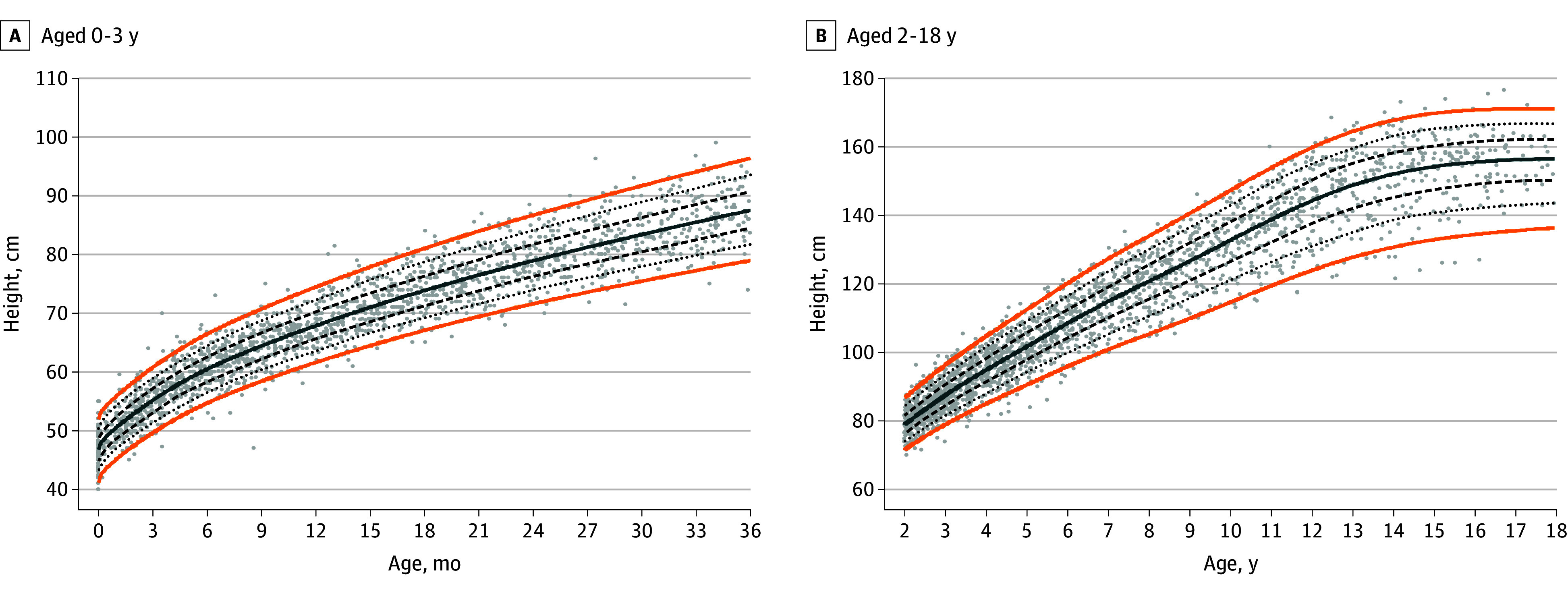
Condition-Specific Height-for-Age Charts for Girls With Alagille Syndrome Orange lines indicate the third and 97th percentiles; solid blue line, the 50th percentile; dashed lines, the 25th and 75th percentiles; dotted lines, the 10th and 90th percentiles; and dots, observations.

The generalized additive models for location scale and shape–generated WFA charts for boys and girls are provided in Figure 1 and Figure 2, respectively, and for HFA for boys and girls in Figure 3 and Figure 4, respectively. The LMS parameters are provided in eTables 3 and 4 in Supplement 1. A BCCG distribution was associated with the best balance between smoothness and adequate local fit for all percentiles, without overcomplicating the model by adjusting for kurtosis. For the WFA charts, 9855 observations (boys, 5740 [58.2%]; girls, 4115 [41.8%]) were used, and for the HFA charts, 8464 observations (boys, 4901 [57.9%]; girls, 3563 [42.1%]) were used. Overlay plots of the condition-specific WFA and HFA growth charts and the corresponding US Centers for Disease Control and Prevention (CDC) growth charts (eFigure 3 in Supplement 1) showed a marked difference in growth pattern between children with ALGS and their native liver compared with US children with typical development. At age 3 years, the 50th CDC weight percentile is near the 90th ALGS-specific percentile for boys and between the 90th and 97th ALGS-specific percentiles for girls. The 50th ALGS-specific percentile lies between the third and 10th CDC percentiles for boys and coincides with the third CDC percentile for girls. The difference attenuates slightly as age progresses for boys, with the 50th ALGS-specific weight percentile between the 10th and 25th CDC weight percentiles at age 18 years, but not for girls (on the third CDC percentile). Similarly at 3 years, the 50th CDC height percentile lies between the 90th and 97th ALGS-specific percentiles for boys and coincides with the 97th percentile for girls. The 50th ALGS-specific height percentile is on the third CDC percentile for boys and below it for girls, while at age 18 years, it is near the 25th CDC percentile for boys and below the third CDC percentile for girls.

Sensitivity analyses were performed for the third, 10th, 25th, 50th, 75th, 90th, and 97th percentiles (eFigure 4 in Supplement 1). The percentiles produced by the models were based on the first or last observation overlap for all weight and height percentiles. Differences between the weight curves based on the interval selection and the interval-weighted selection were minimal. Differences between the height curves were minor for both methods in the first 3 years but more pronounced at older ages, with the interval-weighted curve showing some shrinkage. A grid examination of the modeled weight and height percentiles is presented in eTable 5 in Supplement 1, showing a 1% or less difference between the estimated and observed percentage of observations for nearly all percentiles.

### Performance of the Growth Charts in Children With at Least 10 Years of Follow-Up

For 98 boys and 81 girls, weight data over a time span of at least 10 years were available, with a median of 17 weight measurements (IQR, 10-24 weight measurements). In this subcohort, 15 children (8.4%) received a liver transplant and 5 (2.8%) died. A linear mixed model including age (years), liver transplant, death, and sex confirmed a statistically significant downward trend in weight *z* score over time (−0.01 [95% CI, −0.02 to −0.01]), even after accounting for the significant interaction between time and liver transplant (*z* score, −0.03 [95% CI, −0.05 to −0.01]).

Ten-year follow-up height data were available for 65 girls and 72 boys, with a median of 17 height measurements (IQR, 13-23 height measurements). A total of 11 children (8.0%) received a liver transplant and 5 (3.6%) died. A linear mixed model including age (years), liver transplant, death, and sex confirmed a statistically significant downward trend in height *z* score over time (−0.03 [95% CI, −0.03 to −0.02), even after accounting for the significant interaction between time and liver transplant (*z* score, −0.05 [95% CI, −0.07 to −0.03]); the association was more pronounced in girls (interaction, 0.01 [95% CI, 0.00-0.02]). The linear mixed models are presented in eTable 6 in Supplement 1.

## Discussion

This case series leveraged an international, multicenter rare disease registry to develop the first condition-specific growth charts for children with ALGS to our knowledge. The inclusion of children from all continents may enable the global use of these growth charts. We observed that weight and height growth of children with ALGS is markedly different than in children with typical development. These differences are already apparent at birth, with more than half of the children being born SGA. Syndrome-specific growth charts have been developed for several other genetic conditions, such as Down syndrome,^20,21^ Turner syndrome,^22^ or Prader-Willi syndrome.^23,24^ The robustness of our percentiles was reflected in the sensitivity analyses, which showed similar percentiles using different statistical approaches. Furthermore, they performed well when examined longitudinally in a subgroup of children with at least 10 years of follow-up data available. These growth charts may better inform clinicians on how their individual patient’s growth and nutritional status compares with other children with ALGS and better assess the value of new therapies for enhancing growth in these children.

Children with ALGS already present with a weight and length deficit at birth, as 52% in our study were born SGA. We found a median birth weight and length of 2.8 kg and 48.0 cm, respectively, for boys born at full term and 2.6 kg and 47.0 cm, respectively, for girls born at full term. These findings correspond with those of Quiros-Tejeira et al,^25^ who described similar deficits in 26 children with ALGS born at full term, and further support the theory that height deficit in ALGS is at least partially associated with the underlying genetic mutation. Low birth anthropometrics could also be used as an extra argument in the differential diagnosis from biliary atresia, which can be challenging in early infancy^26^ but does not typically include low birth weight^27,28^ and includes less pronounced length deficits.^28^

Both height and weight differed most in early life between US children with typical development and children with ALGS and their native liver captured in the GALA study. While this difference attenuated later in adolescence, a final height deficit remained compared with individuals without ALGS. The estimated height corresponding to the 50th percentile was 171.5 cm for boys and 156.5 cm for girls on the condition-specific growth charts vs 176 cm and 163 cm, respectively, on the CDC growth charts. For boys, these findings might represent an underestimation of their final height as a less pronounced pubertal growth spurt was observed based on visual inspection of the condition-specific growth charts. Especially in the lower percentiles for boys, an ongoing increasing trend was still present, suggesting that their pubertal growth spurt had not yet completed. Girls, on the other hand, seemed to have an earlier and less pronounced growth spurt compared with the CDC growth charts. There are few data published on pubertal timing in children with ALGS. Kindler et al^29^ reported the individual pubertal scores of 9 girls and 1 boy with ALGS in their study on cortical and trabecular bone status, in which the only boy had a Tanner stage 3 at age 17 years, which is considerably later compared with US reference data.^30^ Significantly delayed bone ages (all 2 SDs less than the mean) were also reported in 9 US children with ALGS, but no further information on their sex or pubertal status was available.^25^ However, we cannot exclude a possible modeling effect explaining the increasing trend in boys given the paucity of growth data after age 18 years. Additional data from an adolescent and adult population are needed to investigate this increasing trend.

Apart from the genetic component, GH insensitivity has also been proposed as one of the mechanisms of short stature in children with ALGS^11,12^ as evidenced by high circulating GH concentrations and low serum insulin-like growth factor 1 values in children with ALGS and short stature, even after the administration of recombinant human GH.^12^ A lack of response to GH therapy was also reported in 2 children with ALGS by Quiros-Tejeira et al.^25^ Growth in infancy is, however, mainly driven by nutritional factors, while the effect of GH deficiency on stature usually only becomes evident in late infancy, and slow growth may not even appear before age 2 to 3 years.^31^ Nutritional status, liver disease, and genetics may be the most important determinants early in life. Growth analysis of 266 children with cholestatic ALGS and available height measurements showed a negative association of total bilirubin levels with growth.^3^ Pruritus or increased serum bile salts may also negatively impact growth, as an increase in height and weight *z* scores was observed after partial external biliary diversion in a small prospective cohort of 7 children (2 with ALGS) with intractable pruritus and cholestatic liver disease.^32^ An increase in height *z* scores was also noted in children with ALGS and intractable pruritus treated with maralixibat, an apical, sodium-dependent, bile acid transport inhibitor.^9^ Both studies lacked a control group, however, making it difficult to distinguish between a true effect on growth and the natural height evolution in children with ALGS, as was observed in our cohort. The majority of the cohort included in this study had cholestasis as a clinical feature, and therefore, the growth patterns reported describe a liver-predominant phenotype. It is possible that individuals with a cardiac or kidney-predominant phenotype with minimal or no liver involvement may present with a different growth pattern.

### Limitations

This study had a couple limitations. First, the growth charts reflect how children with ALGS and their native liver grow, but they do not necessarily reflect optimal growth for children with ALGS. These growth charts help provide perspective for clinicians treating children with ALGS. They reflect the different growth patterns that are observed and could help avoid unnecessary nutritional interventions when a child is tracking along these percentiles. A nutritional intervention should be the result of a nutritional assessment that requires a comprehensive approach that integrates anthropometrics, nutrition-focused history, and physical examination.^33^ Approximately 1 in 3 children in our cohort had a nutritional intervention in the form of supplemental enteral or parenteral support.

Second, a substantial number of children may have received other forms of nutritional support that were not registered in the GALA study, such as oral nutritional support, calorie-dense meals, or monomodal supplementation of additional fats under the form of medium-chain triglycerides, which suggests that the nutritional status of these children was suboptimal and may have affected their growth. Disease status also plays a role, as liver transplant was a significant estimator in our models for the condition-specific weight and height *z* scores. Furthermore, an analysis of 91 children with ALGS showed more pronounced catch-up growth, especially in the first 2 years after liver transplant, than in children transplanted for biliary atresia. However, the final height *z* scores of the children with ALGS remained substantially lower than those of children with biliary atresia.^10^ Similarly, catch-up in weight and height *z* scores was seen in a cohort of children with ALGS who underwent liver transplant, approaching values of a comparison group of children with ALGS with their native liver. For both groups, *z* scores remained well below 0,^25^ suggesting that growth deficit might be, at least partially, considered inherent to the genetic variation. The use of these condition-specific growth charts may identify fewer children with growth failure, especially in early infancy. Growth failure was listed as the primary indication for liver transplant in 54% in a previous report of the GALA cohort.^13^ As the median age of transplant was 2.8 years, the use of these growth charts may change the interpretation of growth failure in the future as this may now be interpreted in reference to other patients with ALGS and their native liver. When using these condition-specific growth charts, clinicians should expect that children on average will have a 0.1 decrease in weight *z* score and of 0.3 in height *z* score over the span of 10 years. Such changes may not be sufficient to trigger an intervention in clinical practice and suggest that the assessment of disease-related malnutrition is not always straightforward in children. For example, even though low weight and height *z* scores are frequently reported in children with ALGS, studies that have assessed body composition mostly noted normal fat stores.^2,4,7,8,11^ Reducing the nutritional status of a child (regardless of the underlying condition) to merely a weight or height may be an oversimplification of the nutritional assessment, which is why we opted to also include other parameters, such as the mid–upper-arm circumference and skinfolds in the Excel calculator in Supplement 2. The importance of an anthropometric assessment that goes beyond simply measuring weight and height was recently highlighted in a position paper by the clinical malnutrition special interest group of the European Society for Pediatric Gastroenterology, Hepatology and Nutrition.^34^ This holistic approach to nutritional assessment that includes the use of our growth charts in addition to anthropometrics may improve data for decision-making around listing children for liver transplant. We anticipate that these growth curves may help identify children who need a transplant and perhaps reassure clinicians in the case of children whose growth is tracking appropriately. The future development of a danger zone of low WFA, analogous to that created for children with cerebral palsy,^35^ independently associated with a poor outcome, such as liver transplant or death, may also help guide decision-making for more aggressive nutritional interventions in ALGS.

## Conclusions

In this case series, we developed condition-specific growth charts for children with ALGS. Weight and height of children with ALGS were markedly different than in children with typical development. These growth charts may better inform clinicians on how their individual patient’s growth and nutritional status compares with other children with ALGS as well as lead to a better appreciation of placebo-controlled drug trials that might have an impact on growth in these children.

## Notes

Supplement 1. **eTable 1.** Different Characteristics Between Both Datasets **eTable 2.** Number of Weight and Height Data Points per Age Interval **eTable 3.** LMS Tables for Weight and Age **eTable 4. **LMS Tables for Height and Age **eTable 5. **Distribution of the Modeled Weight and Height for Age Centiles **eTable 6. **Linear Mixed Model of WFA and HFA ALGS-Specific *z* Scores **eFigure 1.** Study Flowchart **eFigure 2.** Birth Weight and Length of Children With ALGS Born at Full Term **eFigure 3.** ALGS-Specific vs CDC Weight and Length/Height for Age Centiles in Boys and Girls **eFigure 4.** Sensitivity Analyses for Weight and Height for Age Centiles in Boys and Girls

Supplement 2.
Excel Macro

Supplement 3.
Data Sharing Statement
