# Error in Nonbyline Author List

**DOI:** 10.1001/jamanetworkopen.2025.55321

**Published:** 2025-12-26

In the Original Article titled “Condition-Specific Growth Charts for Children With Alagille Syndrome,”^1^ published November 24, 2025, the nonbyline author list was not placed correctly. This article has been corrected.^1^
