# Supplementary material for: Condition-Specific Growth Charts for Children With Alagille Syndrome
Source: JAMA Netw Open. 2025 Nov 24;8(11):e2545294. doi: 10.1001/jamanetworkopen.2025.45294 (PMC12645329; doi:10.1001/jamanetworkopen.2025.45294)
Supplement: Supplement 1. — eTable 1. Different Characteristics Between Both Datasets eTable 2. Number of Weight and Height Data Points per Age Interval eTable 3. LMS Tables for Weight and Age eTable 4. LMS Tables for Height and Age eTable 5. Distribution of the Modeled Weight and Height for Age Centiles eTable 6. Linear Mixed Model of WFA and HFA ALGS-Specific z Scores eFigure 1. Study Flowchart eFigure 2. Birth Weight and Length of Children With ALGS Born at Full Term eFigure 3. ALGS-Specific vs CDC Weight and Length/Height for Age Centiles in Boys and Girls eFigure 4. Sensitivity Analyses for Weight and Height for Age Centiles in Boys and Girls [file jamanetwopen-e2545294-s001.pdf]

Supplemental Online Content

Huysentruyt K, Vandriel SM, Roelants M, et al; Global Alagille Alliance (GALA) Study Group. Condition-specific growth charts for children with Alagille syndrome. *JAMA Netw Open*. 2025;8(11):e2545294. doi:10.1001/jamanetworkopen.2025.45294

eTable 1. Different Characteristics Between Both Datasets

eTable 2. Number of Weight and Height Data Points per Age Interval

eTable 3. LMS Tables for Weight and Age

eTable 4. LMS Tables for Height and Age

eTable 5. Distribution of the Modeled Weight and Height for Age Centiles

eTable 6. Linear Mixed Model of WFA and HFA ALGS-Specific *z* Scores

eFigure 1. Study Flowchart

eFigure 2. Birth Weight and Length of Children With ALGS Born at Full Term

eFigure 3. ALGS-Specific vs CDC Weight and Length/Height for Age Centiles in Boys and Girls

eFigure 4. Sensitivity Analyses for Weight and Height for Age Centiles in Boys and Girls

This supplemental material has been provided by the authors to give readers additional information about their work.

© 2025 Huysentruyt K et al. *JAMA Network Open*.

eTable 1. Different Characteristics Between Both Datasets

	Original dataset (term children)	Balanced dataset (term children)
**Weight measurements**		
Total observations	14632	9855
Patients	1204	1204
Observations per patient:		
- Min – max	1-291	1-52
- Median (Q1;Q3)	6 (2;14)	5 (2;12)
Follow-up time (y)		
- Min - max	0-21	0-20
- Median (Q1;Q3)	2.4 (0.2;6.3)	2.4 (0.3;6.3)
**Length/height measurements**		
Total observations	10595	8463
Patients	1106	1106
Observations per patient:		
- Min – max	1-142	1-44
- Median (Q1;Q3)	5 (2;13)	5 (2;11)
Follow-up time (y)		
- Min - max	0-21	0-20
- Median (Q1;Q3)	1.9 (0.0;5.7)	2.3 (0.3;6.3)

Balanced dataset was created by taking only the first weight and height measurement available per patient in the defined time intervals (see methods section for further explanation on the time intervals)

© 2025 Huysentruyt K et al. *JAMA Network Open*.

eTable 2. Number of Weight and Height Data Points per Age Interval

eTable 2a. Number of weight data points per age interval

Birth		0-1 mo		1-2 mo		2-3 mo		3-4 mo		4-5 mo	
M	F	M	F	M	F	M	F	M	F	M	F
363	284	88	57	131	92	185	114	125	89	129	85
**5-6 mo**		**6-7 mo**		**7-8 mo**		**8-9 mo**		**9-10 mo**		**10-11mo**	
M	F	M	F	M	F	M	F	M	F	M	F
126	81	142	100	119	77	127	77	109	79	116	68
**11-12 mo**		**12-13 mo**		**13-14 mo**		**14-15 mo**		**15-16 mo**		**16-17 mo**	
M	F	M	F	M	F	M	F	M	F	M	F
99	61	121	66	110	61	101	62	98	60	90	51
**17-18 mo**		**18-19 mo**		**19-20 mo**		**20-21 mo**		**21-22 mo**		**22-23 mo**	
M	F	M	F	M	F	M	F	M	F	M	F
79	53	90	64	83	54	77	54	87	46	71	46
**23-24 mo**		**24-26 mo**		**27-29 mo**		**30-32 mo**		**33-35 mo**		**36-41 mo**	
M	F	M	F	M	F	M	F	M	F	M	F
61	47	159	116	138	109	134	101	130	93	197	134
**42-47 mo**		**48-53 mo**		**54-59 mo**		**60-65 mo**		**66-71 mo**		**72-77 mo**	
M	F	M	F	M	F	M	F	M	F	M	F
185	135	160	128	149	112	125	85	110	82	113	81
**78-83 mo**		**84-89 mo**		**90-95 mo**		**96-101 mo**		**102-107 mo**		**108-113 mo**	
M	F	M	F	M	F	M	F	M	F	M	F
102	70	93	64	79	68	88	68	80	58	74	56
**114-119 mo**		**120-125 mo**		**126-131 mo**		**132-137 mo**		**138-143 mo**		**144-149 mo**	
M	F	M	F	M	F	M	F	M	F	M	F
59	50	66	50	64	52	51	54	55	50	49	46
**150-155 mo**		**156-161 mo**		**162-167 mo**		**168-173 mo**		**174-179 mo**		**180-185 mo**	
M	F	M	F	M	F	M	F	M	F	M	F
48	49	42	38	38	34	32	39	36	28	31	27
**186-191 mo**		**192-197 mo**		**198-203 mo**		**204-209 mo**		**210-215 mo**		**≥216 mo**	
M	F	M	F	M	F	M	F	M	F	M	F
27	22	21	25	22	17	13	12	14	14	29	20

© 2025 Huysentruyt K et al. *JAMA Network Open*.

eTable 2b. Number of height data points per age interval

Birth		0-1 mo		1-2 mo		2-3 mo		3-4 mo		4-5 mo	
M	F	M	F	M	F	M	F	M	F	M	F
174	284	68	57	100	92	134	114	102	89	104	85
**5-6 mo**		**6-7 mo**		**7-8 mo**		**8-9 mo**		**9-10 mo**		**10-11mo**	
M	F	M	F	M	F	M	F	M	F	M	F
99	81	114	100	100	77	101	77	87	79	92	68
**11-12 mo**		**12-13 mo**		**13-14 mo**		**14-15 mo**		**15-16 mo**		**16-17 mo**	
M	F	M	F	M	F	M	F	M	F	M	F
76	61	94	66	91	61	78	62	86	60	73	51
**17-18 mo**		**18-19 mo**		**19-20 mo**		**20-21 mo**		**21-22 mo**		**22-23 mo**	
M	F	M	F	M	F	M	F	M	F	M	F
67	53	77	64	70	54	67	54	72	46	60	46
**23-24 mo**		**24-26 mo**		**27-29 mo**		**30-32 mo**		**33-35 mo**		**36-41 mo**	
M	F	M	F	M	F	M	F	M	F	M	F
51	47	147	116	122	109	121	101	119	93	178	134
**42-47 mo**		**48-53 mo**		**54-59 mo**		**60-65 mo**		**66-71 mo**		**72-77 mo**	
M	F	M	F	M	F	M	F	M	F	M	F
174	135	158	128	145	112	122	85	97	82	108	81
**78-83 mo**		**84-89 mo**		**90-95 mo**		**96-101 mo**		**102-107 mo**		**108-113 mo**	
M	F	M	F	M	F	M	F	M	F	M	F
101	70	93	64	72	68	86	68	73	58	72	56
**114-119 mo**		**120-125 mo**		**126-131 mo**		**132-137 mo**		**138-143 mo**		**144-149 mo**	
M	F	M	F	M	F	M	F	M	F	M	F
56	50	64	50	63	52	50	54	54	50	48	46
**150-155 mo**		**156-161 mo**		**162-167 mo**		**168-173 mo**		**174-179 mo**		**180-185 mo**	
M	F	M	F	M	F	M	F	M	F	M	F
48	49	40	38	38	34	31	39	35	28	31	27
**186-191 mo**		**192-197 mo**		**198-203 mo**		**204-209 mo**		**210-215 mo**		**≥216 mo**	
M	F	M	F	M	F	M	F	M	F	M	F
26	22	20	25	21	17	12	12	14	14	25	20

© 2025 Huysentruyt K et al. *JAMA Network Open*.

eTable 3. LMS Tables for Weight for Age

Age (y)	L		M		S		Age (y)	L		M		S	
	♂	♀	♂	♀	♂	♀		♂	♀	♂	♀	♂	♀
0.08	0.38	-0.15	3.31	3.16	0.162	0.165	8.5	-0.73	-0.94	22.84	22.81	0.169	0.191
0.25	0.27	-0.24	4.62	4.17	0.156	0.161	9	-0.82	-0.99	24.05	24.12	0.176	0.197
0.5	0.19	-0.01	5.90	5.37	0.149	0.157	9.5	-0.89	-1.00	25.33	25.53	0.183	0.203
0.75	0.16	0.20	6.83	6.29	0.144	0.154	10	-0.93	-0.99	26.70	27.05	0.191	0.208
1	0.14	0.29	7.65	7.10	0.140	0.149	10.5	-0.94	-0.97	28.18	28.67	0.198	0.214
1.33	0.15	0.27	8.66	8.08	0.136	0.143	11	-0.93	-0.95	29.78	30.35	0.205	0.219
1.67	0.18	0.15	9.47	8.92	0.133	0.137	11.5	-0.90	-0.91	31.49	32.08	0.211	0.224
2	0.25	0.03	10.21	9.67	0.131	0.133	12	-0.86	-0.88	33.32	33.83	0.216	0.228
2.5	0.39	-0.08	11.22	10.69	0.130	0.131	12.5	-0.82	-0.84	35.25	35.57	0.221	0.232
3	0.52	-0.12	12.15	11.70	0.129	0.134	13	-0.78	-0.79	37.26	37.27	0.224	0.235
3.5	0.55	-0.11	13.08	12.65	0.129	0.138	13.5	-0.75	-0.74	39.34	38.93	0.226	0.238
4	0.51	-0.11	13.98	13.57	0.130	0.142	14	-0.74	-0.70	41.46	40.53	0.228	0.241
4.5	0.42	-0.12	14.87	14.46	0.131	0.147	14.5	-0.74	-0.66	43.60	42.08	0.229	0.243
5	0.30	-0.17	15.77	15.39	0.134	0.153	15	-0.76	-0.62	45.75	43.57	0.229	0.244
5.5	0.17	-0.24	16.69	16.35	0.137	0.158	15.5	-0.78	-0.60	47.89	45.02	0.229	0.245
6	0.03	-0.34	17.62	17.32	0.141	0.163	16	-0.81	-0.59	50.02	46.43	0.229	0.246
6.5	-0.12	-0.47	18.58	18.32	0.146	0.169	16.5	-0.85	-0.59	52.11	47.80	0.228	0.247
7	-0.28	-0.62	19.56	19.36	0.151	0.174	17	-0.88	-0.60	54.18	49.13	0.228	0.248
7.5	-0.45	-0.76	20.60	20.45	0.156	0.180	17.5	-0.92	-0.60	56.22	50.44	0.227	0.248
8	-0.60	-0.87	21.70	21.59	0.162	0.185	18	-0.96	-0.61	58.23	51.72	0.227	0.249

© 2025 Huysentruyt K et al. *JAMA Network Open*.

eTable 4. LMS Tables for Height for Age

Age (y)	L		M		S		Age (y)	L		M		S	
	♂	♀	♂	♀	♂	♀		♂	♀	♂	♀	♂	♀
0.08	0.017	1.15	51.19	50.64	0.0547	0.0564	8.5	2.10	2.41	123.26	123.74	0.0574	0.0629
0.25	0.98	0.83	56.44	55.02	0.0489	0.0534	9	1.92	2.53	125.82	126.71	0.0596	0.0634
0.5	1.51	0.67	62.45	60.51	0.0462	0.0517	9.5	1.74	2.65	128.36	129.74	0.0622	0.0638
0.75	1.82	0.55	66.25	64.47	0.0453	0.0508	10	1.59	2.76	130.97	132.81	0.0649	0.0641
1	1.97	0.47	69.63	67.90	0.0452	0.0503	10.5	1.47	2.87	133.71	135.86	0.0677	0.0643
1.33	2.03	0.41	73.70	72.00	0.0455	0.0502	11	1.38	2.97	136.62	138.85	0.0704	0.0643
1.67	2.04	0.39	77.28	75.67	0.0462	0.0504	11.5	1.34	3.07	139.71	141.69	0.0727	0.0643
2	2.05	0.42	80.52	78.95	0.0471	0.0508	12	1.34	3.16	142.98	144.32	0.0744	0.0641
2.5	2.15	0.52	84.93	83.42	0.0485	0.0518	12.5	1.40	3.25	146.35	146.71	0.0753	0.0638
3	2.33	0.67	88.88	87.55	0.0497	0.0529	13	1.50	3.34	149.73	148.82	0.0754	0.0635
3.5	2.53	0.84	92.57	91.35	0.0505	0.0540	13.5	1.65	3.42	153.03	150.61	0.0745	0.0630
4	2.70	1.02	96.09	94.91	0.0509	0.0552	14	1.84	3.50	156.16	152.10	0.0729	0.0624
4.5	2.81	1.21	99.51	98.36	0.0511	0.0563	14.5	2.06	3.57	159.06	153.31	0.0707	0.0618
5	2.86	1.39	102.82	101.76	0.0511	0.0574	15	2.31	3.64	161.66	154.27	0.0681	0.0611
5.5	2.86	1.55	106.04	105.19	0.0512	0.0584	15.5	2.57	3.71	163.93	155.01	0.0652	0.0604
6	2.81	1.71	109.15	108.56	0.0516	0.0593	16	2.86	3.77	165.90	155.56	0.0622	0.0596
6.5	2.73	1.86	112.16	111.80	0.0521	0.0601	16.5	3.16	3.83	167.60	155.96	0.0593	0.0588
7	2.61	2.01	115.07	114.92	0.0530	0.0609	17	3.47	3.89	169.07	156.25	0.0565	0.0580
7.5	2.46	2.15	117.88	117.91	0.0542	0.0617	17.5	3.78	3.94	170.33	156.46	0.0538	0.0573
8	2.29	2.28	120.61	120.82	0.0556	0.0623	18	4.10	4.00	171.44	156.60	0.0513	0.0565

© 2025 Huysentruyt K et al. *JAMA Network Open*.

eTable 5. Distribution of the Modeled Weight and Height for Age Centiles

	Weight cohort		Height cohort	
	Boys	Girls	Boys	Girls
% of observations below 3rd centile	2.7	3.3	2.7	2.9
% of observations below 10th centile	9.0	9.0	9.8	10.0
% of observations below 25th centile	24.4	22.8	24.9	24.8
% of observations below 50th centile	50.1	50.4	51.0	50.8
% of observations below 75th centile	75.6	77.2	74.2	74.8
% of observations below 90th centile	90.5	90.4	89.6	90.3
% of observations below 97th centile	96.9	96.7	97.4	97.1

© 2025 Huysentruyt K et al. *JAMA Network Open*.

eTable 6. Linear Mixed Model of WFA and HFA ALGS-Specific *z* Scores

eTable 6a. Linear mixed model of WFA ALGS-specific z scores

Predictor	Estimate	95% CI	p-value
(intercept)	0.06	-0.12;0.24	
Visit age (years)	-0.01	-0.02;-0.01	<0.001
Liver transplant (yes)	-0.10	-0.54;0.34	0.67
Sex (boys)	-0.03	-0.27;0.21	0.81
Died (yes)	-0.72	-1.46;0.03	0.06
Visit age * liver transplant	-0.03	-0.05;-0.01	<0.001

eTable 6b. Linear mixed model of HFA ALGS-specific z scores

Predictor	Estimate	95% CI	p-value
(intercept)	0.15	-0.07;0.37	
Visit age (years)	-0.03	-0.03;-0.02	<0.001
Liver transplant (yes)	-0.41	-0.96;0.14	0.14
Sex (boys)	-0.12	-0.41;0.18	0.45
Died (yes)	-0.87	-1.68;-0.07	0.03
Visit age * liver transplant	-0.05	-0.07;-0.03	<0.001
Visit age * sex (boys)	0.01	0.00;0.02	0.01

© 2025 Huysentruyt K et al. *JAMA Network Open*.

eFigure 1. Study Flowchart

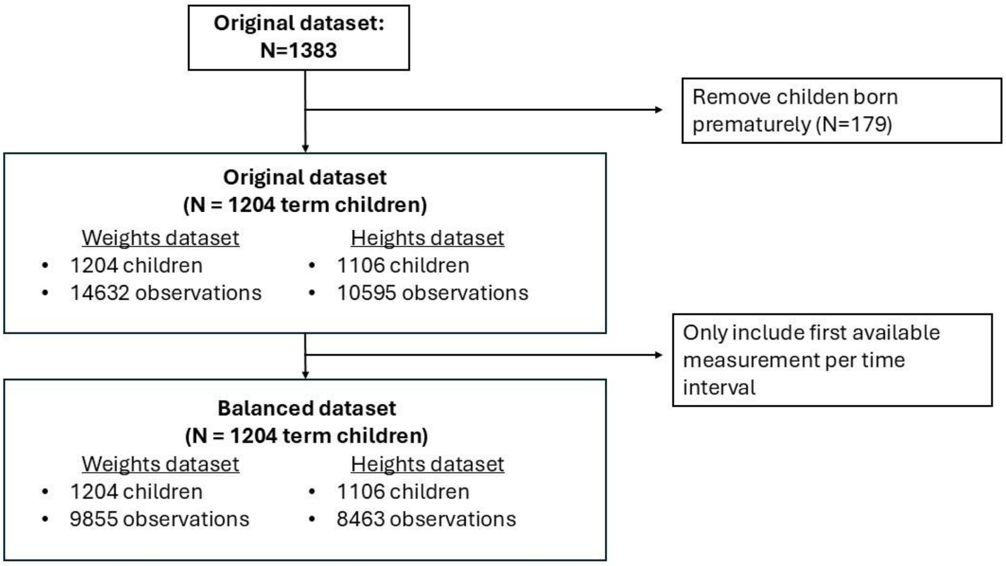

© 2025 Huysentruyt K et al. *JAMA Network Open*.

eFigure 2. Birth Weight and Length of Children With ALGS Born at Full Term

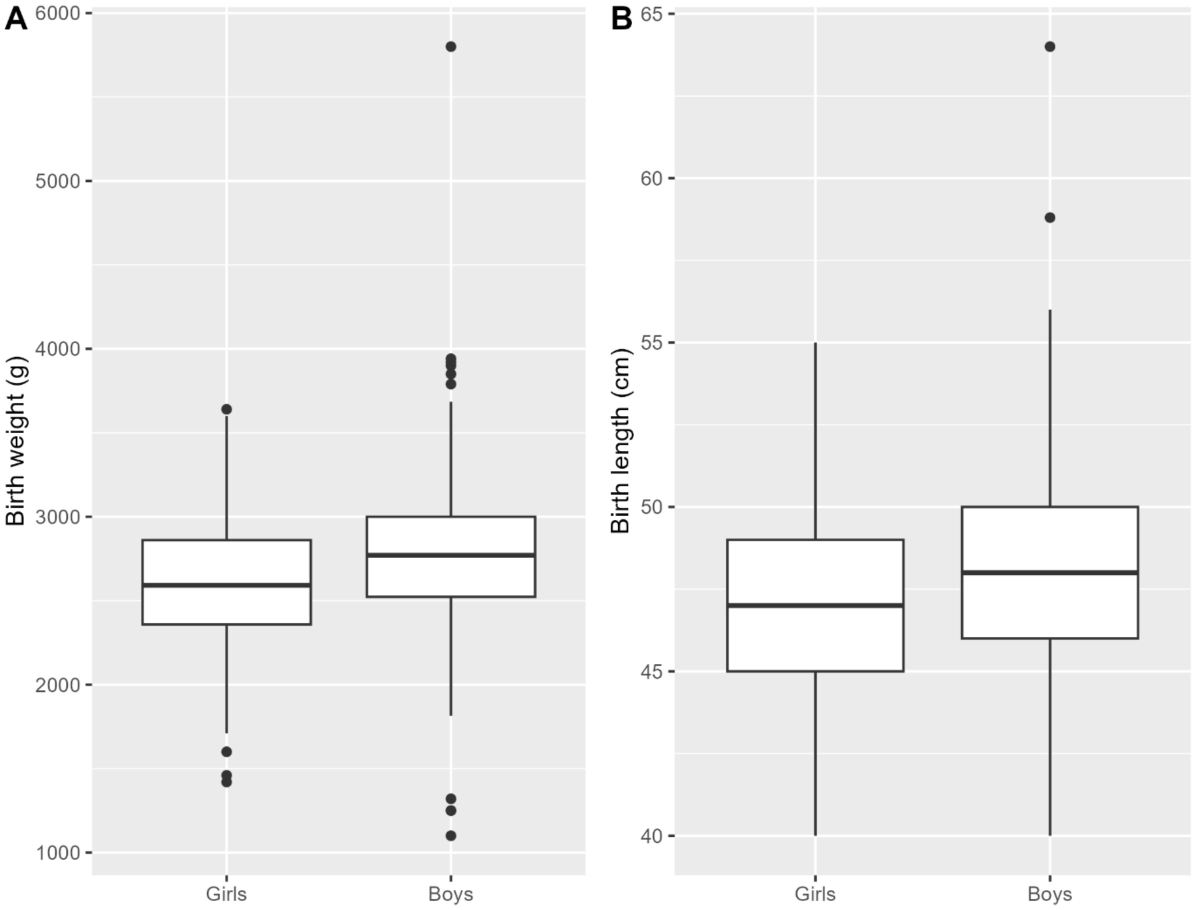

© 2025 Huysentruyt K et al. *JAMA Network Open*.

eFigure 3. ALGS-Specific vs CDC Weight and Length/Height for Age Centiles in Boys and Girls

eFigure 3a. ALGS-specific vs CDC weight for age centiles (boys)

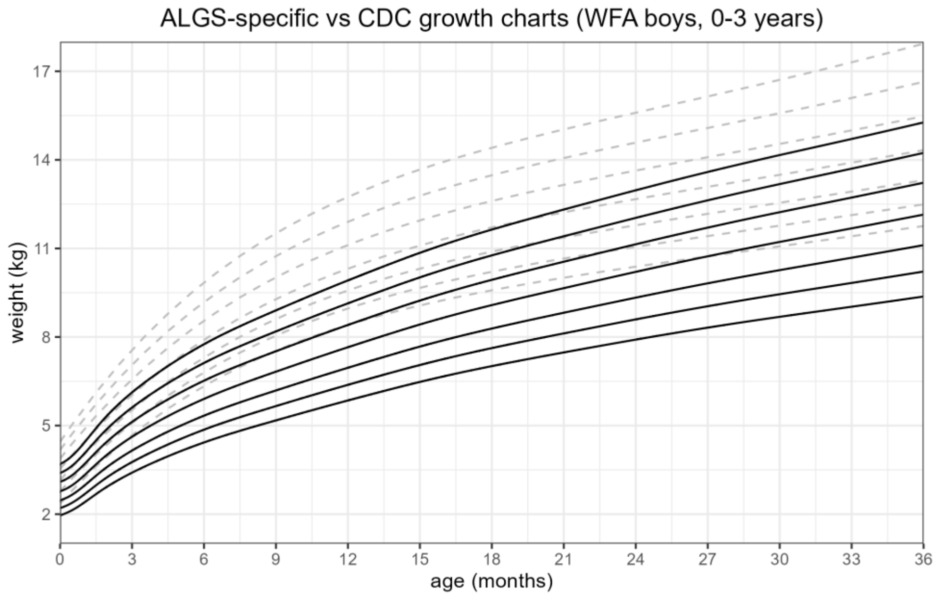

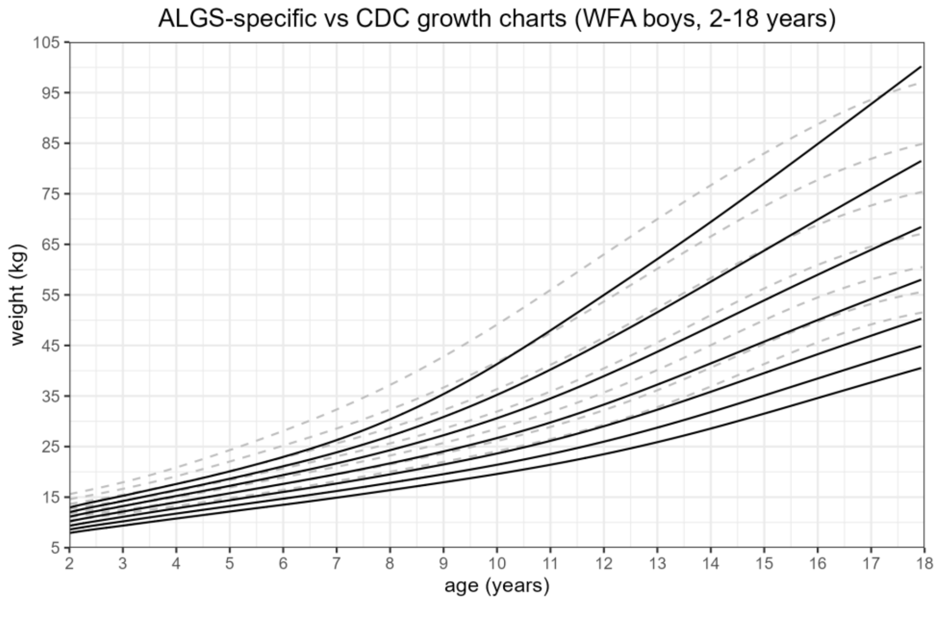

Dashed lines: 3rd, 10th, 25th, 50th, 75th, 90th and 97th CDC centile; full lines: corresponding ALGS-specific centiles

© 2025 Huysentruyt K et al. *JAMA Network Open*.

eFigure 3b. ALGS-specific vs CDC weight for age centiles (girls)

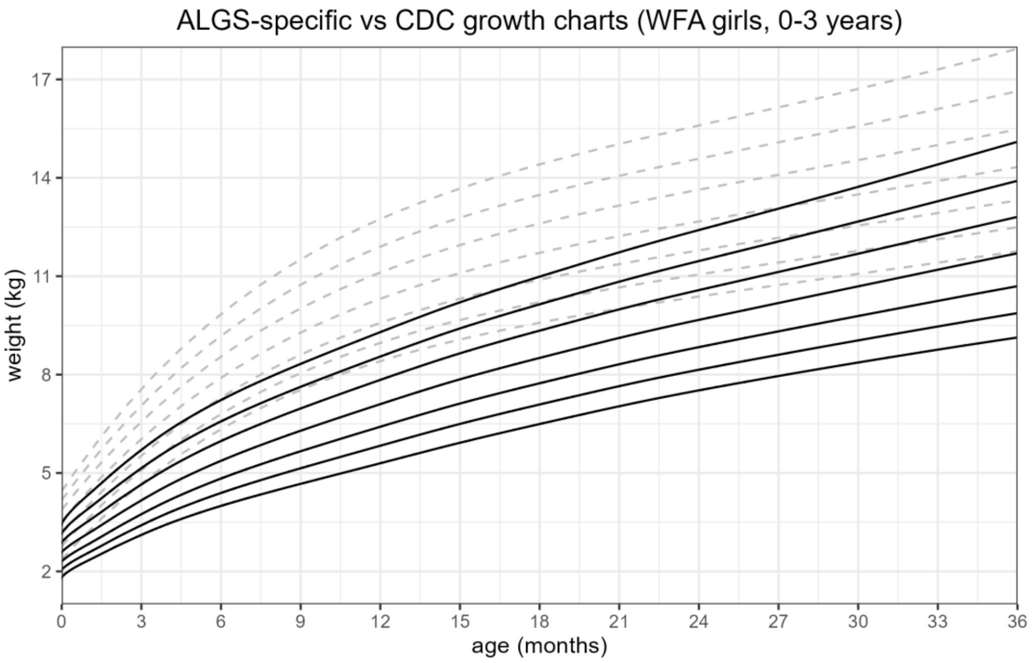

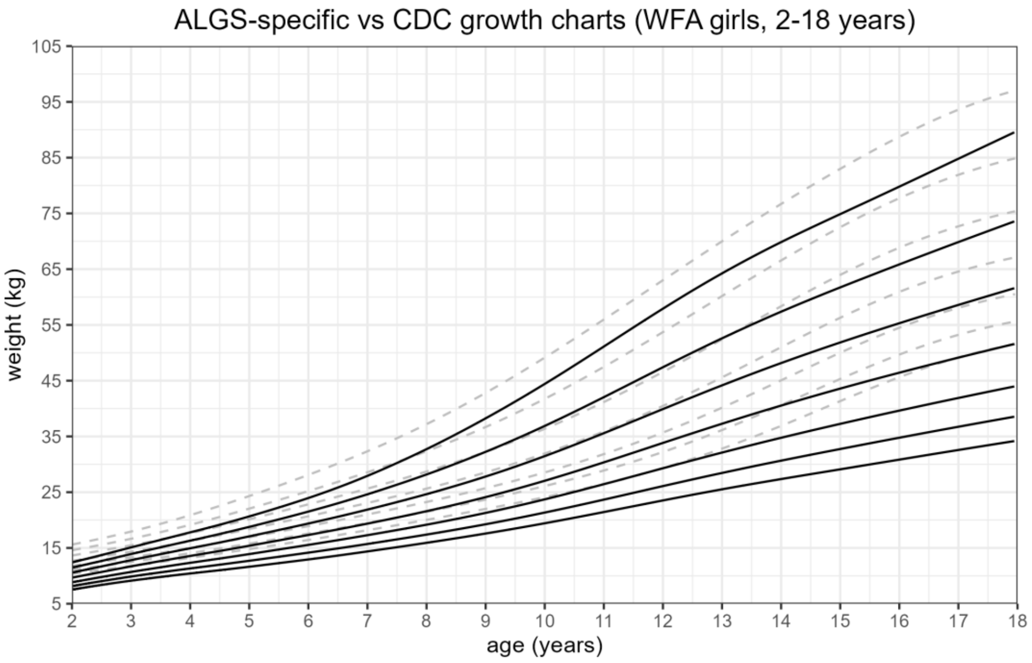

Dashed lines: 3rd, 10th, 25th, 50th, 75th, 90th and 97th CDC centile; full lines: corresponding ALGS-specific centiles

© 2025 Huysentruyt K et al. *JAMA Network Open*.

eFigure 3c. ALGS-specific vs CDC length/height for age centiles (boys)

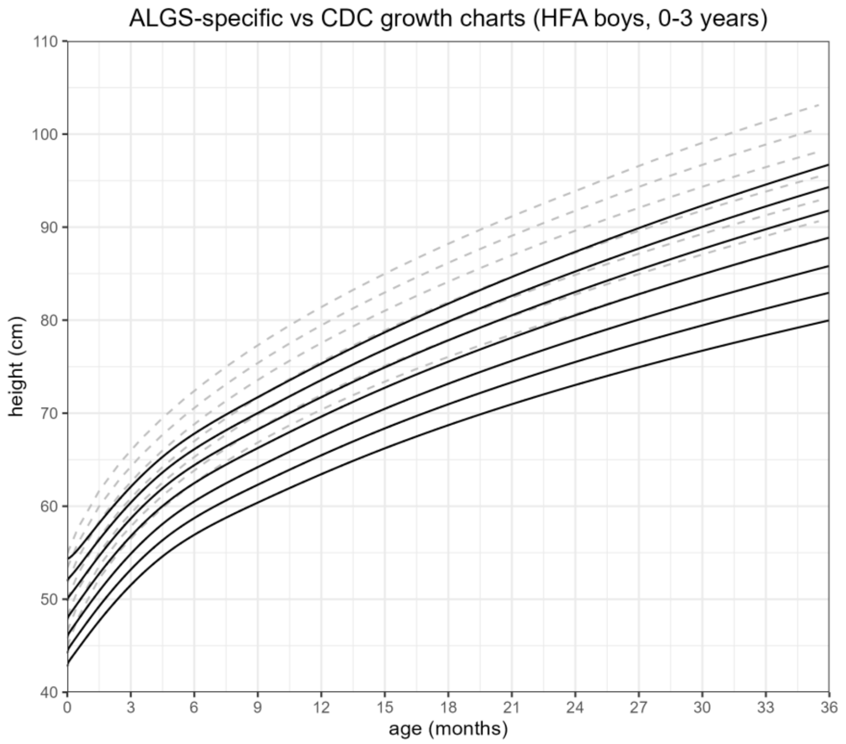

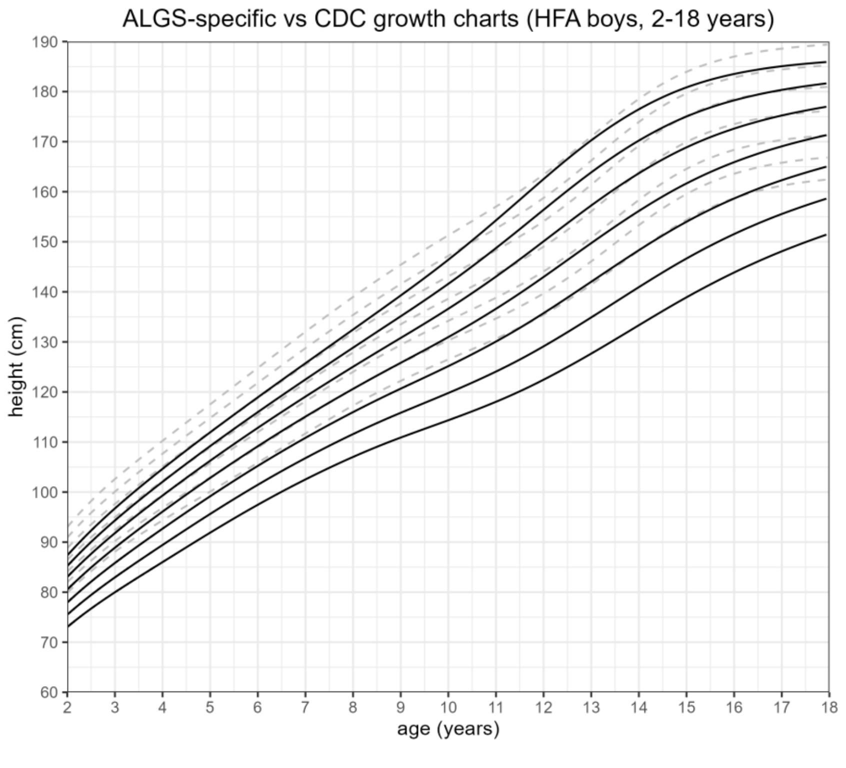

Dashed lines: 3rd, 10th, 25th, 50th, 75th, 90th and 97th CDC centile; full lines: corresponding ALGS-specific centiles

© 2025 Huysentruyt K et al. *JAMA Network Open*.

eFigure 3d. ALGS-specific vs CDC length/height for age centiles (girls)

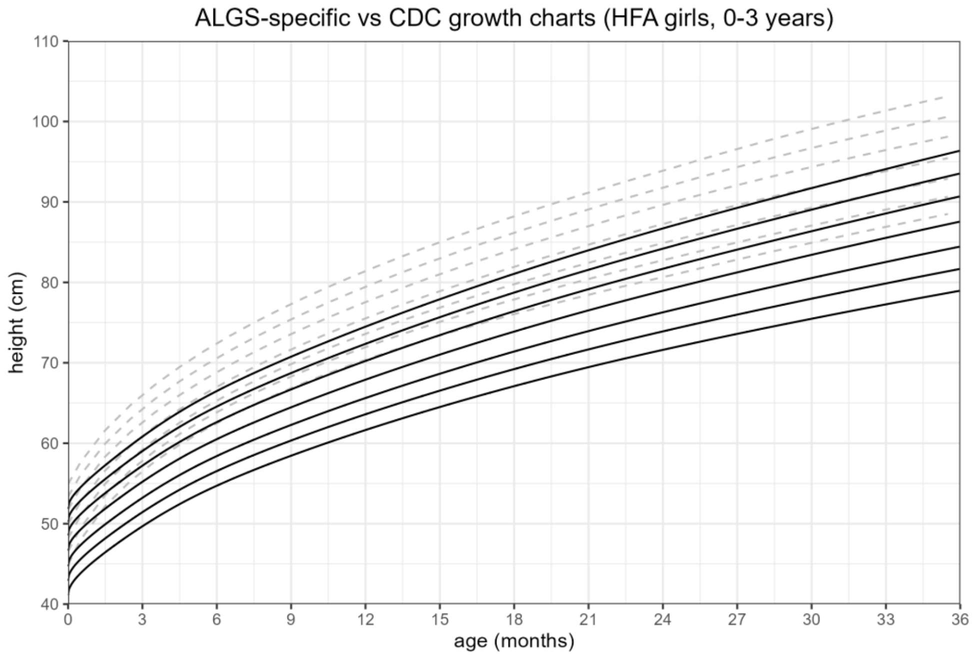

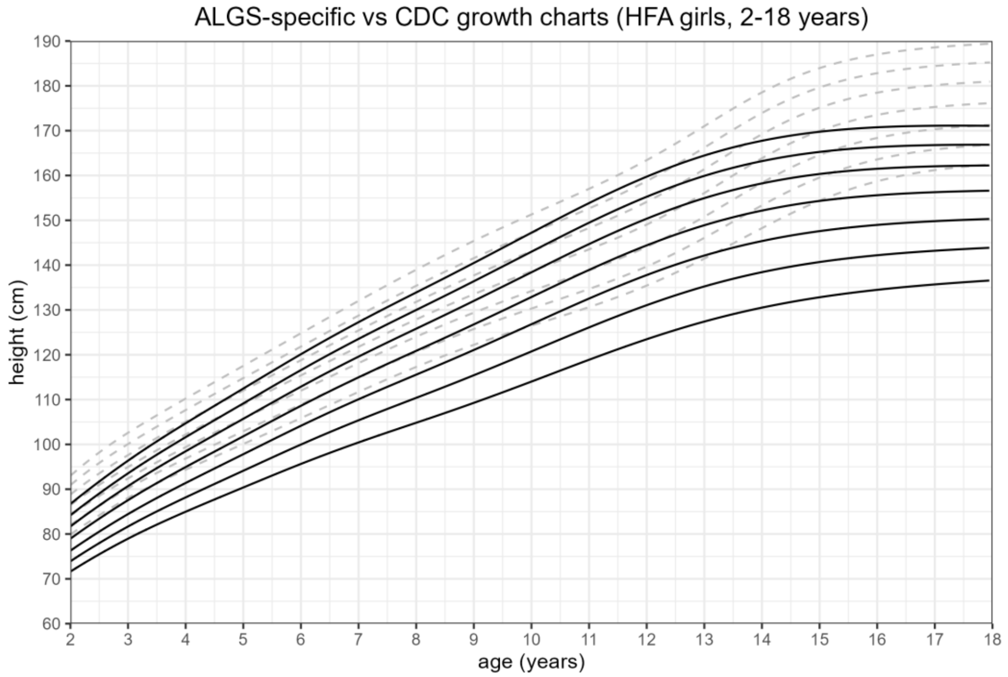

Dashed lines: 3rd, 10th, 25th, 50th, 75th, 90th and 97th CDC centile; full lines: corresponding ALGS-specific centiles

© 2025 Huysentruyt K et al. *JAMA Network Open*.

eFigure 4. Sensitivity Analyses for Weight and Height for Age Centiles in Boys and Girls

eFigure 4a. Sensitivity analysis for weight for age centiles (boys)

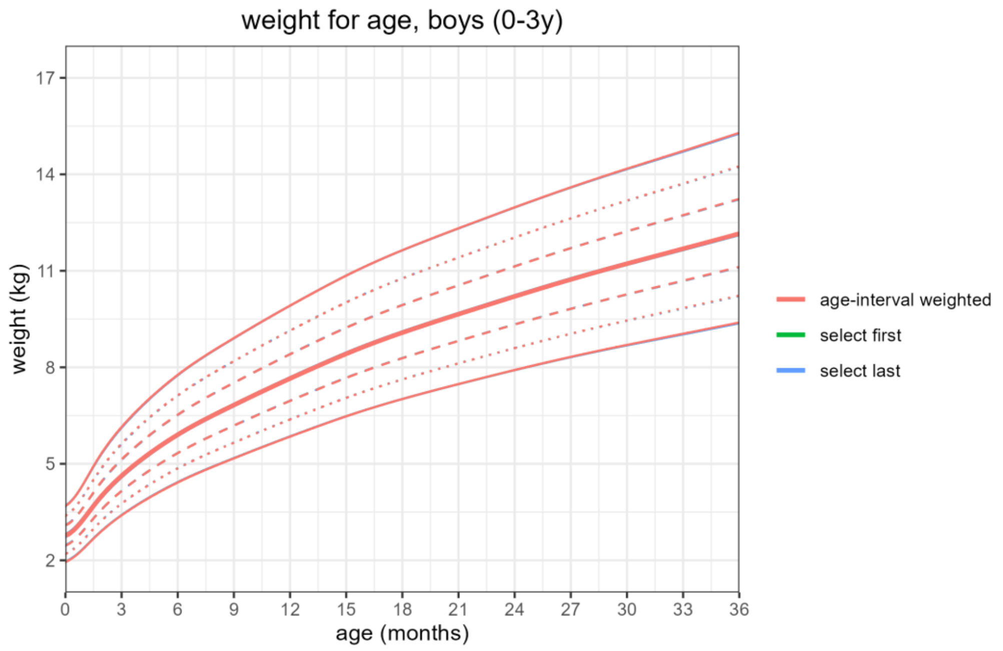

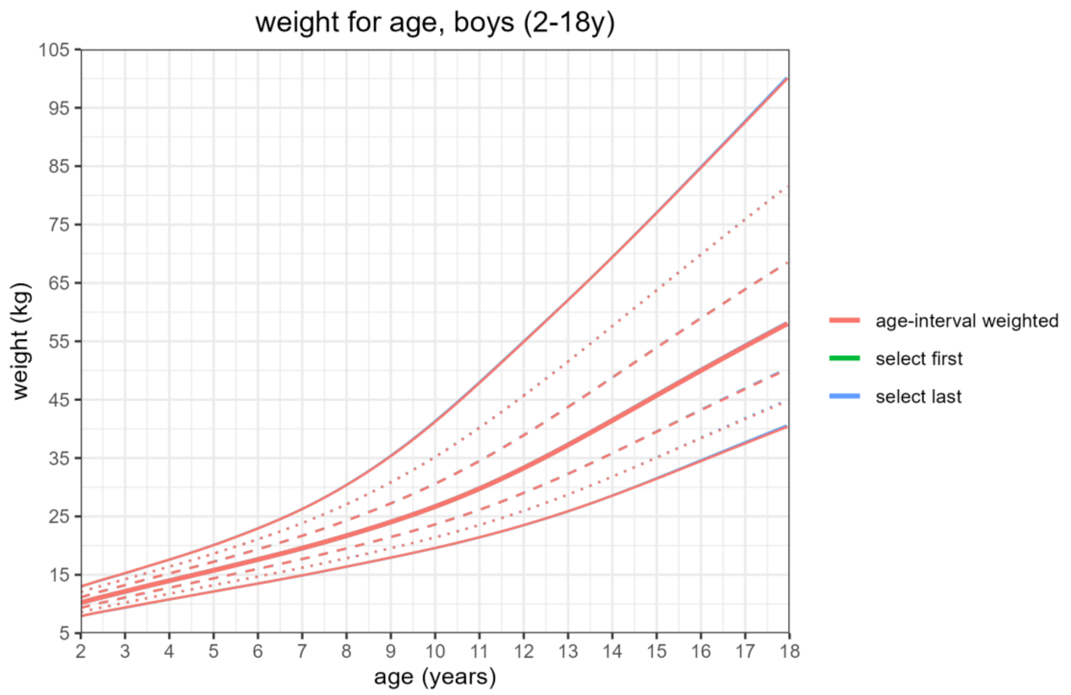

© 2025 Huysentruyt K et al. *JAMA Network Open*.

eFigure 4b. Sensitivity analysis for weight for age centiles (girls)

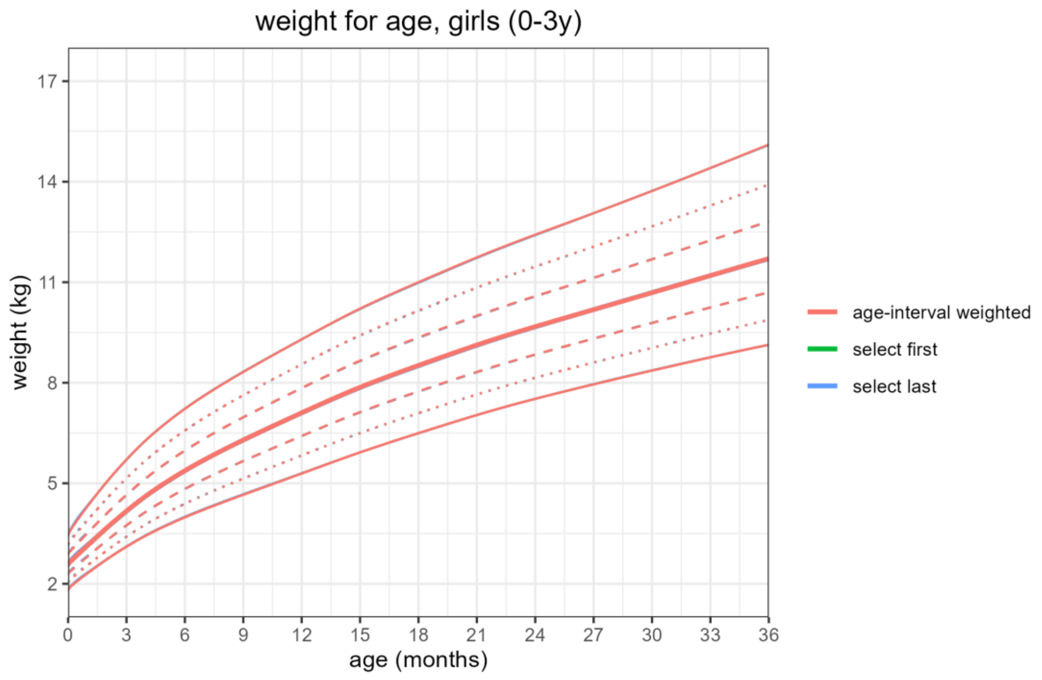

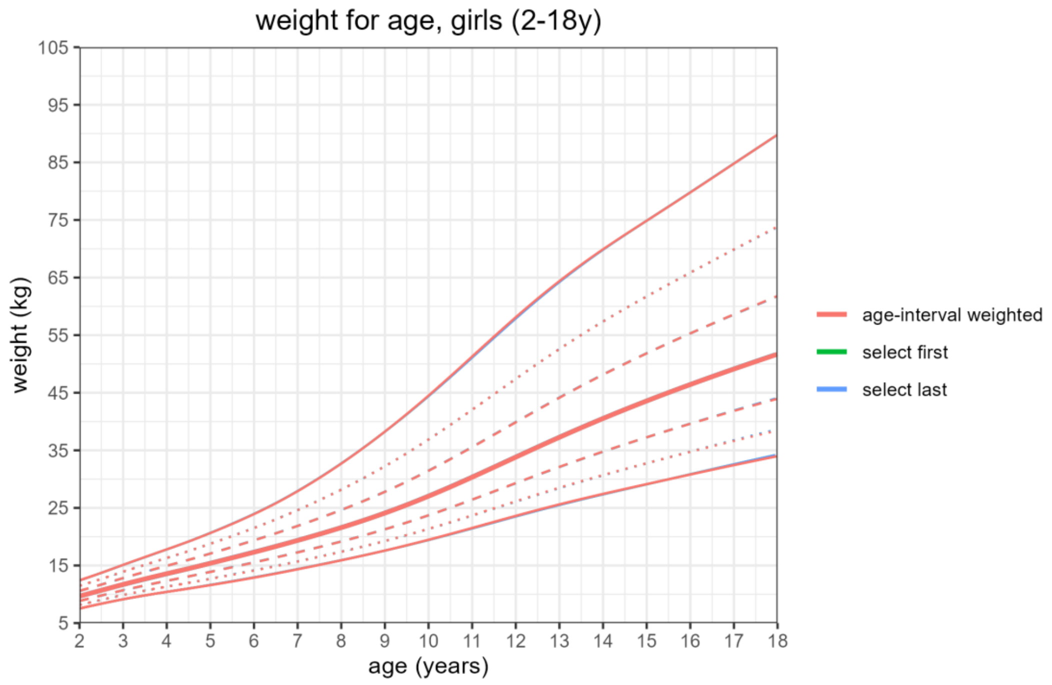

© 2025 Huysentruyt K et al. *JAMA Network Open*.

eFigure 4c. Sensitivity analysis for height for age centiles (boys)

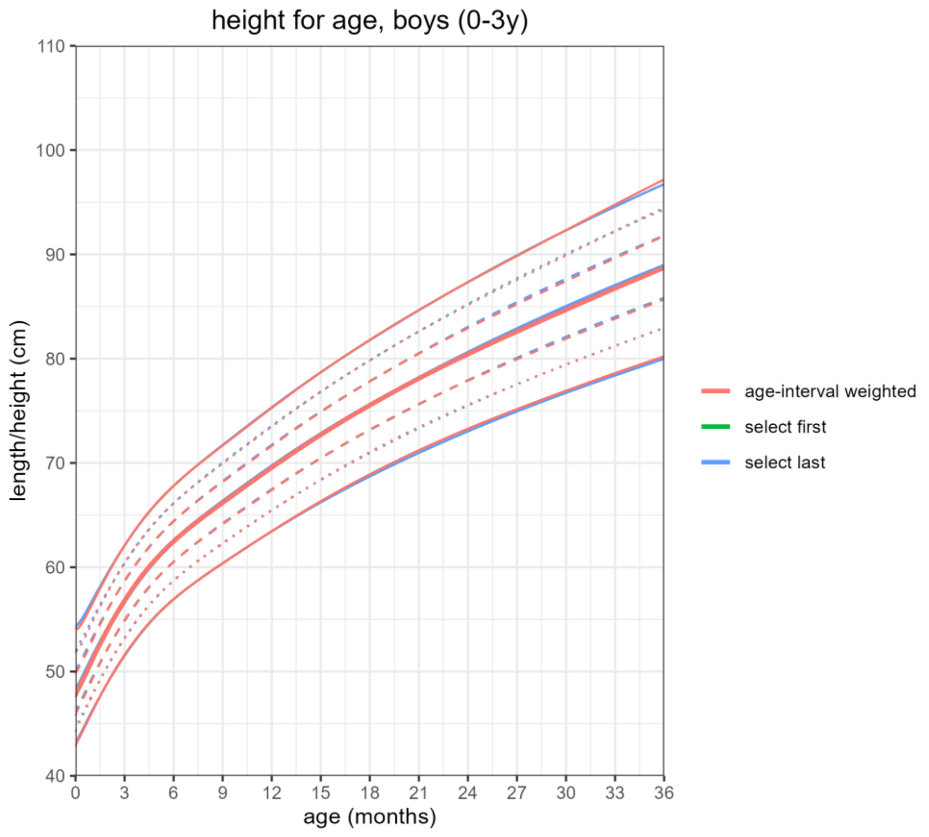

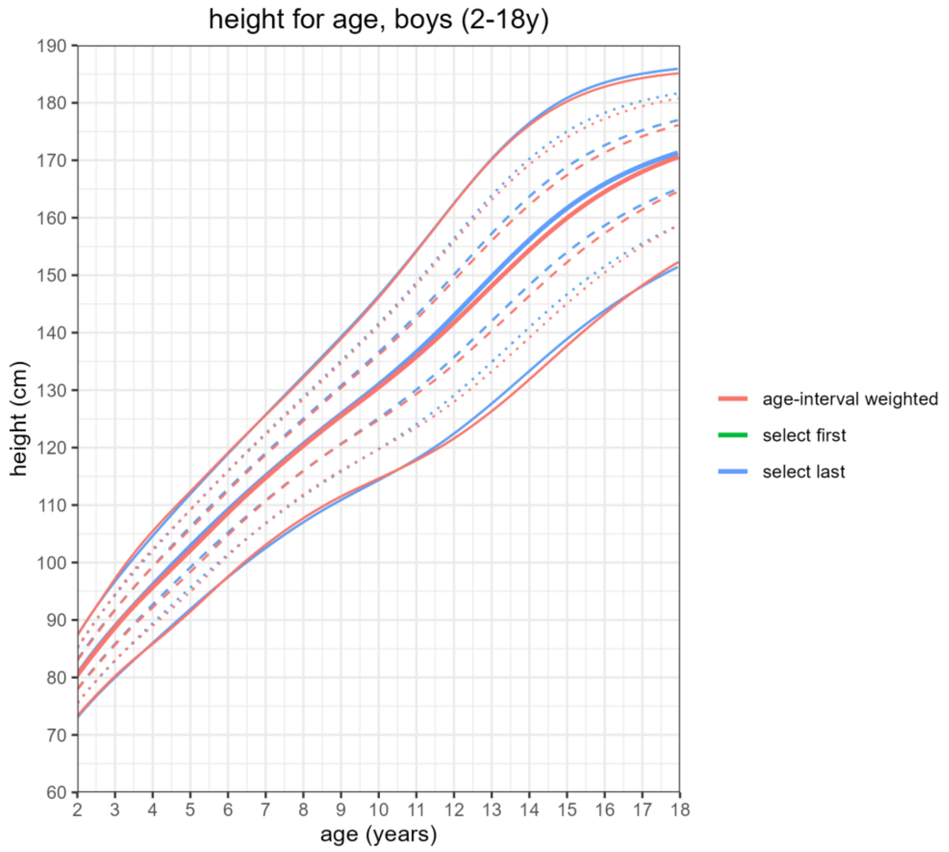

© 2025 Huysentruyt K et al. *JAMA Network Open*.

eFigure 4d. Sensitivity analysis for height for age centiles (girls)

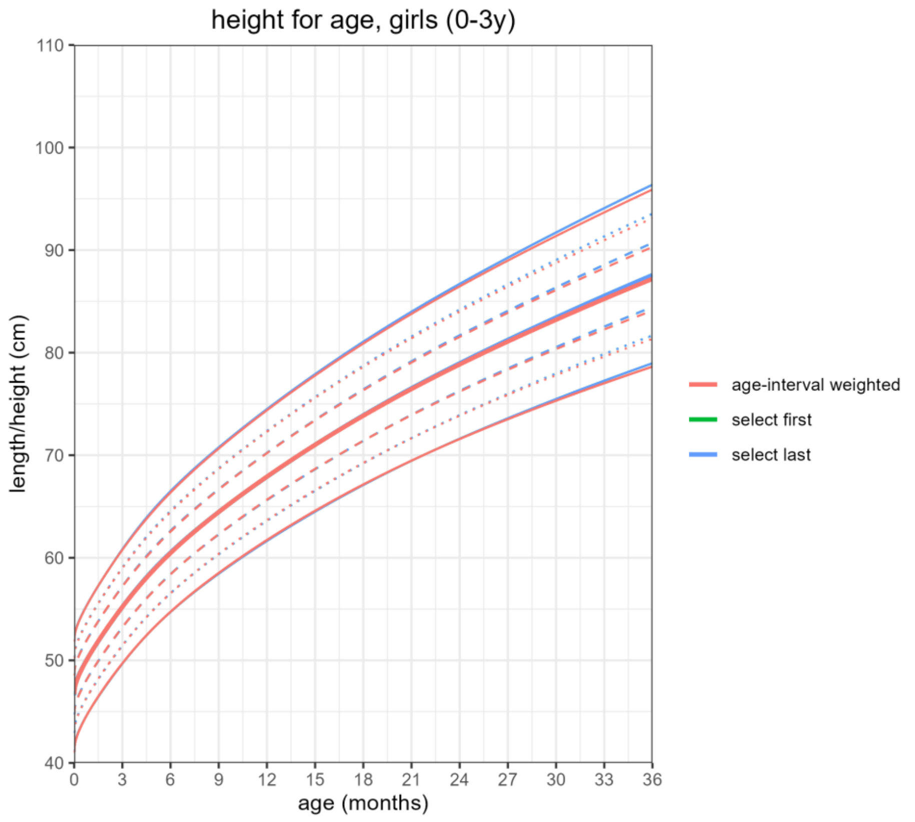

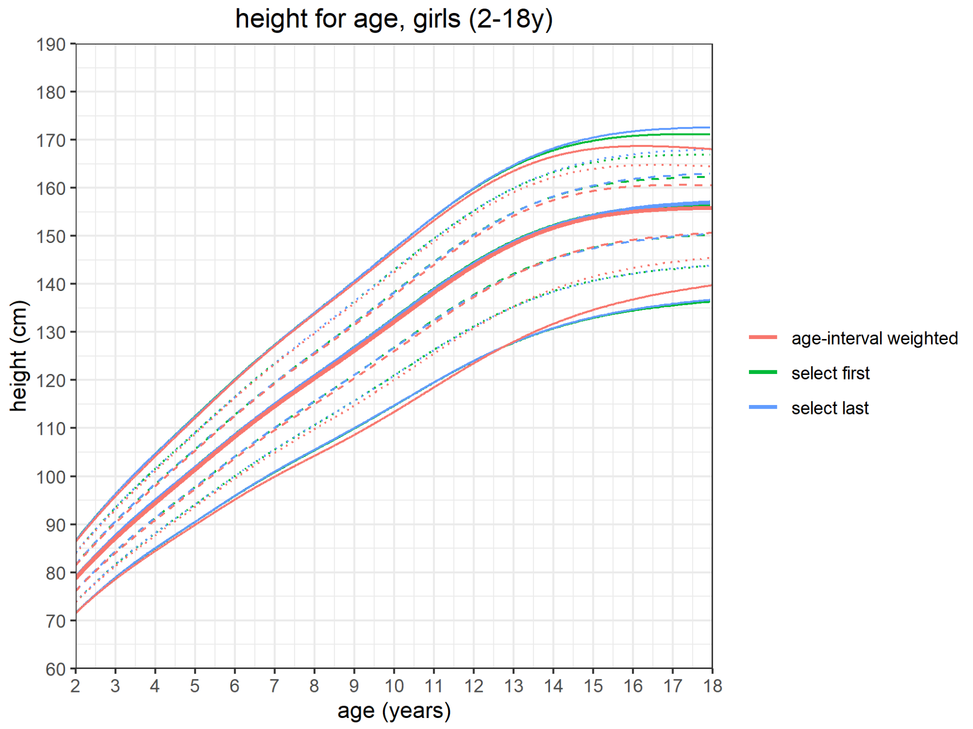

© 2025 Huysentruyt K et al. *JAMA Network Open*.
