# Supplementary material for: Condition-Specific Growth Charts for Children With Alagille Syndrome
Source: JAMA Netw Open. 2025 Nov 24;8(11):e2545294. doi: 10.1001/jamanetworkopen.2025.45294 (PMC12645329; doi:10.1001/jamanetworkopen.2025.45294)
Supplement: Supplement 3. — Data Sharing Statement [file jamanetwopen-e2545294-s003.pdf]

Data Sharing Statement

Data
Data available: No
. The data are not publicly available due to privacy or ethical restrictions.
